# Lipids, Lipoproteins, and Metabolites and Risk of Myocardial Infarction and Stroke

**DOI:** 10.1016/j.jacc.2017.12.006

**Published:** 2018-02-13

**Authors:** Michael V. Holmes, Iona Y. Millwood, Christiana Kartsonaki, Michael R. Hill, Derrick A. Bennett, Ruth Boxall, Yu Guo, Xin Xu, Zheng Bian, Ruying Hu, Robin G. Walters, Junshi Chen, Mika Ala-Korpela, Sarah Parish, Robert J. Clarke, Richard Peto, Rory Collins, Liming Li, Zhengming Chen, China Kadoorie Biobank Collaborative Group

**Affiliations:** a Clinical Trial Service Unit & Epidemiological Studies Unit (CTSU), Nuffield Department of Population Health, University of Oxford, Oxford, United Kingdom; b Medical Research Council Population Health Research Unit (MRC PHRU) at the University of Oxford, Nuffield Department of Population Health, University of Oxford, Oxford, United Kingdom; c National Institute for Health Research Oxford Biomedical Research Centre, Oxford University Hospital, Oxford, United Kingdom; d Chinese Academy of Medical Sciences, Beijing, China; e Liuyang CDC, Changsha, China; f NCDs Prevention and Control Department, Zhejiang CDC, Hangzhou, China; g National Center for Food Safety Risk Assessment, Beijing, China; h Computational Medicine, Faculty of Medicine, University of Oulu and Biocenter Oulu, Oulu, Finland; i Medical Research Council Integrative Epidemiology Unit at the University of Bristol, Bristol, United Kingdom; j Population Health Science, Bristol Medical School, University of Bristol, Bristol, United Kingdom; k NMR Metabolomics Laboratory, School of Pharmacy, University of Eastern Finland, Kuopio, Finland; l Systems Epidemiology, Baker Heart and Diabetes Institute, Melbourne, Victoria, Australia; m Department of Epidemiology and Preventive Medicine, School of Public Health and Preventive Medicine, Faculty of Medicine, Nursing and Health Sciences, the Alfred Hospital, Monash University, Melbourne, Victoria, Australia; n Department of Global Health, School of Public Health, Peking University, Beijing, China

**Keywords:** epidemiology, etiology, intracerebral hemorrhage, ischemic stroke, metabolomics, myocardial infarction, BMI, body mass index, CABG, coronary artery bypass grafting, CHD, coronary heart disease, CI, confidence interval, CKB, China Kadoorie Biobank, CVD, cardiovascular disease, HDL-C, high-density lipoprotein cholesterol, IDL, intermediate-density lipoprotein, IHD, ischemic heart disease, IS, ischemic stroke, LDL-C, low-density lipoprotein cholesterol, MI, myocardial infarction, NMR, nuclear magnetic resonance, PCI, percutaneous coronary intervention, RCT, randomized controlled trial, SBP, systolic blood pressure, TG, triglycerides, VLDL, very low-density lipoprotein

## Abstract

**Background:**

Blood lipids are established risk factors for myocardial infarction (MI), but uncertainty persists about the relevance of lipids, lipoprotein particles, and circulating metabolites for MI and stroke subtypes.

**Objectives:**

This study sought to investigate the associations of plasma metabolic markers with risks of incident MI, ischemic stroke (IS), and intracerebral hemorrhage (ICH).

**Methods:**

In a nested case-control study (912 MI, 1,146 IS, and 1,138 ICH cases, and 1,466 common control subjects) 30 to 79 years of age in China Kadoorie Biobank, nuclear magnetic resonance spectroscopy measured 225 metabolic markers in baseline plasma samples. Logistic regression was used to estimate adjusted odds ratios (ORs) for a 1-SD higher metabolic marker.

**Results:**

Very low-, intermediate-, and low-density lipoprotein particles were positively associated with MI and IS. High-density lipoprotein (HDL) particles were inversely associated with MI apart from small HDL. In contrast, no lipoprotein particles were associated with ICH. Cholesterol in large HDL was inversely associated with MI and IS (OR: 0.79 and 0.88, respectively), whereas cholesterol in small HDL was not (OR: 0.99 and 1.06, respectively). Triglycerides within all lipoproteins, including most HDL particles, were positively associated with MI, with a similar pattern for IS. Glycoprotein acetyls, ketone bodies, glucose, and docosahexaenoic acid were associated with all 3 diseases. The 225 metabolic markers showed concordant associations between MI and IS, but not with ICH.

**Conclusions:**

Lipoproteins and lipids showed similar associations with MI and IS, but not with ICH. Within HDL particles, cholesterol concentrations were inversely associated, whereas triglyceride concentrations were positively associated with MI. Glycoprotein acetyls and several non–lipid-related metabolites associated with all 3 diseases.

Observational studies and randomized controlled trials have demonstrated that low-density lipoprotein cholesterol (LDL-C) is one of the most important modifiable and causal risk factors for coronary heart disease (CHD) and ischemic stroke (IS) 1, 2, 3, 4. The relationship of LDL-C with risk of intracerebral hemorrhage (ICH) is less clear 4, 5, and the role of other lipids (e.g., high-density lipoprotein cholesterol [HDL-C] and triglycerides [TG]) in the etiology of cardiovascular disease (CVD) subtypes is not so well established (1).

Conventional measures of circulating lipids (e.g., LDL-C and HDL-C) fail to distinguish among the size, density, or concentrations and compositions of lipoproteins, which may have contrasting relevance to risk of CVD (6). For example, conventional measures of HDL-C include the sum of cholesterol carried in HDL particles, but ignore their composition (including TG and phospholipids), particle size, and subclass concentration. Likewise, the usual measures of TG reflect the summation of TG carried across the various lipoproteins; just as for cholesterol, TG may show divergent relationships with vascular disease when transported in different lipoprotein particles. Therefore, a more detailed investigation of the association of individual lipoprotein particle subclasses and lipid-related traits with risk of CHD and stroke subtypes could be informative.

Quantitative nuclear magnetic resonance (NMR) metabolomics provides detailed measurements of lipoprotein particle concentrations and their associated lipids categorized by particle size, and additional metabolic markers (7). The approach has been used in several population-based epidemiological and genetic studies to date 6, 7, 8, 9, 10, 11, but no study has yet comprehensively investigated the associations of these traits with different CVD subtypes in the same study population. The present study examined the associations of plasma lipoprotein particles and other metabolic markers with risk of incident MI, IS, and ICH in a nested case-control study within the China Kadoorie Biobank (CKB).

## Methods

### Study population

The CKB is a prospective cohort of 512,891 Chinese adults 30 to 79 years of age at enrollment, recruited between 2004 and 2008 from 10 (5 urban, 5 rural) geographically defined areas, as previously described (12). For each participant, detailed information was collected at baseline and at periodic resurveys (5% random subset) by questionnaire, including sociodemographic and lifestyle (e.g., smoking, alcohol intake) factors and medical history (including use of certain specific medications such as statins), and physical measurements (e.g., blood pressure and anthropometry). Participants provided a 10 ml nonfasting blood sample (with time since last meal recorded) that was separated into 1 buffy coat and 3 plasma aliquots. Long-term follow-up of CKB participants was achieved by electronic linkage, using unique personal identification numbers, to established death and morbidity (for cancer, CHD, stroke, and diabetes) registries and to a nationwide health insurance system, which provides coded disease diagnoses (International Classification of Diseases-10th Revision) and procedures (e.g., coronary artery bypass grafting, percutaneous coronary intervention) for each episode of hospitalization. Medical records for all hospitalizations (and a subset of all deaths) due to vascular diseases have been reviewed systematically and independently by clinicians in Oxford and China to minimize misclassification.

### Nested case-control study

A subset of 4,662 individuals was selected for the metabolomics study from a larger nested case-control study of stroke and CHD subtypes comprising ∼23,000 CKB individuals, which included genome-wide array data and standard clinical biochemistry analysis. Cases included in the metabolomics study had an incident fatal or nonfatal event coded as International Classification of Diseases-10th Revision: I21-23 for MI (n = 912); I63 or I69.3 for IS (n = 1,146); and I61 or I69.1 for ICH (n = 1,138), at the censoring date of January 1, 2015, and were selected from the youngest of the available cases at the time of event. Common control subjects (n = 1,466) were selected from among control subjects in the larger study and were frequency matched, where possible, to the combined cases by age, sex, and area. All cases and control subjects had no history of self-reported prior doctor-diagnosed CHD, stroke, transient ischemic attack, and cancer, and were not using statin therapy at baseline.

### Measurement of NMR metabolomics and clinical chemistry

Baseline plasma samples were thawed and subaliquoted at the Wolfson Laboratory (University of Oxford, United Kingdom), and 100-μl aliquots were shipped on dry ice to the Brainshake Laboratory at Kuopio, Finland, for NMR spectroscopy. Of the 4,662 individuals with baseline NMR spectroscopy measurements, 137 had measurements conducted in duplicate. A high-throughput targeted NMR metabolomics platform 7, 11, 13 was used to generate spectra from which 225 lipid and other metabolic measures were simultaneously quantified, either as absolute concentrations of each metabolic measure or as ratios. Cases and control subjects were analyzed in random order and laboratory staff were blinded to case or control status.

Plasma concentrations of 8 traits (total cholesterol, LDL-C, HDL-C, TG, apolipoprotein B, apolipoprotein A1, albumin, and creatinine) that were measured by NMR spectroscopy were also quantified in samples from the same 4,662 participants using standard clinical chemistry assays at the Wolfson Laboratory (AU 680 clinical chemistry analyzers, Beckman Coulter [UK] Ltd., Wycombe, United Kingdom) using manufacturers’ reagents, calibrators, and settings.

### Statistical analysis

To facilitate comparisons between metabolic measures within and across assays, traits were standardized (i.e., values of each metabolic marker were divided by its standard deviation). We calculated Pearson’s correlation coefficients of the 8 biochemical parameters measured by both NMR spectroscopy and standard clinical chemistry. The associations for traits measured by both NMR spectroscopy and clinical chemistry with risk of MI were assessed using logistic regression adjusted for age (in 5-year categories), sex, fasting time (<8 or ≥8 h), region (10 regions), smoking status (never, ex-regular, occasional, current regular), and educational attainment (no formal or primary school, middle or high school, technical school or college or university). Treating fasting time as a continuous trait and adjusting the associations for both fasting time and the square of fasting time had no material effect on the associations. Technical replication (repeated measurement of the same sample) was assessed through coefficients of variation, expressed as a percentage, for individuals that had duplicate measures for NMR spectroscopy (n = 137).

Logistic regression was used to assess the relationships of all 225 metabolic measures by NMR spectroscopy with risks of MI, IS, and ICH separately, using the same adjustments as mentioned previously. For each measure, adjusted odds ratios (ORs) and 95% confidence intervals (CIs) per 1-SD higher metabolic measure were estimated. In subsequent sensitivity analyses, additional adjustments for the associations with disease risk were made for systolic blood pressure (SBP) and body mass index (BMI).

To account for the large number of highly correlated metabolic measures, we used a false discovery rate correction (14) and interpreted false discovery rate–corrected p values <0.05 as providing evidence against the null hypothesis. To test the added discriminatory power of these metabolic measures, we calculated the area under the receiver-operating characteristic curve for 2 models: model 1 included risk factors included in established risk prediction algorithms (age, sex, smoking, SBP, BMI, type 2 diabetes, and total cholesterol/HDL-C ratio) and model 2 included the same covariates as in model 1 and in addition included 18 principal components explaining 95% of the variance in the 225 metabolic markers. All analyses were conducted using R version 3.3.1 (R Project for Statistical Computing, Vienna, Austria).

## Results

### Characteristics of individuals in the nested case-control study

Among the 4,662 participants with metabolomics measurements, the mean age of stroke cases was similar to control subjects (Table 1), but the mean age of MI cases was older (IS 42 years of age, ICH 47 years of age, MI 52 years of age vs. control subjects 45 years of age). Likewise, the proportion of women among stroke cases (IS 54%; ICH 52%) was similar to that of control subjects (52%), but the proportion was lower among MI cases (40%). Cases had higher SBP (MI mean 141 ± 26 mm Hg; IS 135 ± 25 mm Hg; ICH 151 ± 29 mm Hg) than did control subjects (127 ± 18 mm Hg), but levels of overall and central adiposity, height, and physical activity were similar between cases and control subjects (Table 1). Cases were more likely to have self-reported diabetes at baseline (MI 11%, IS 7%, ICH 6% vs. control subjects 3%) and poor self-rated health (MI 16%, IS 13%, ICH 14% vs. control subjects 9%).

**Table 1:** Baseline Characteristics of Participants in the Nested Case-Control Study of Vascular Disease

	Control Subjects (n = 1,466)	Myocardial Infarction (n = 912)	Ischemic Stroke (n = 1,146)	Intracerebral Hemorrhage (n = 1,138)
Age and socioeconomic factors				
Age, yrs	45.0 ± 8.3	52.1 ± 7.1	42.3 ± 6.1	47.0 ± 6.9
Female	52.3	39.6	54.1	52.2
College/university education	5.5	4.1	7.9	2.6
Rural residents	75.1	70.6	60.3	76.5
Lifestyle factors				
Ever regular smoker	31.7	45.8	32.7	32.1
Weekly alcohol drinker	21.7	20.9	24.8	23.5
Physical activity, MET h/day	24.6 ± 14.5	20.4 ± 14.2	22.8 ± 13.8	23.0 ± 14.6
Anthropometry and blood pressure				
Standing height, m	1.6 ± 0.1	1.6 ± 0.1	1.6 ± 0.1	1.6 ± 0.1
BMI, kg/m^2^	23.5 ± 3.3	24.0 ± 3.6	24.6 ± 3.7	24.1 ± 3.6
Waist circumference, cm	79.4 ± 9.5	82.9 ± 10.2	82.6 ± 10.4	81.6 ± 10.2
SBP, mm Hg	126.8 ± 17.8	140.6 ± 25.5	135.0 ± 24.5	150.7 ± 29.0
DBP, mm Hg	76.6 ± 10.5	82.7 ± 13.6	83.3 ± 14.5	89.5 ± 16.2
Lipids measured by clinical chemistry, mg/dl				
LDL cholesterol∗	84.9 ± 27.0	92.7 ± 30.9	88.8 ± 27.0	84.9 ± 27.0
HDL cholesterol	46.3 ± 11.6	45.2 ± 11.6	45.9 ± 11.6	47.1 ± 11.6
Triglycerides	177.0 ± 150.4	194.7 ± 177.0	185.8 ± 150.4	177.0 ± 150.4
Self-reported health and disease				
Poor self-rated health	8.6	16.2	12.5	14.2
Diabetes	3.1	11.4	7.2	6.3
Fasting time, h	4.1 ± 4.1	4.4 ± 4.3	4.6 ± 4.7	4.0 ± 4.1

Values are mean ± SD or %.

BMI = body mass index; DBP = diastolic blood pressure; MET = metabolic equivalent; SBP = systolic blood pressure.

∗ Low-density lipoprotein (LDL) cholesterol was directly measured (and not estimated using the Friedewald equation). To convert high-density lipoprotein (HDL) cholesterol and LDL cholesterol to mmol/l, multiply by 0.0259; to convert triglycerides to mmol/l, multiply by 0.0113.

For clinical chemistry, MI and IS cases had higher mean concentrations of LDL-C (MI 93 ± 31 mg/dl; IS 89 ± 27 mg/dl) than did control subjects (85 ± 27 mg/dl; p < 2.8 × 10^−8^ comparing MI cases with control subjects and IS cases with control subjects), with ICH cases having similar LDL-C values to control subjects. A similar pattern was observed for TG. In contrast, HDL-C concentrations were broadly similar between cases and control subjects. The distribution of fasting times at recruitment to the study was similar for cases and control subjects (Table 1).

### Comparison of traits measured by NMR spectroscopy and clinical biochemistry

Online Table 1 shows the correlation coefficients of lipids (total cholesterol, LDL-C, HDL-C, and TG), apolipoproteins A1 and B, creatinine, and albumin as measured by clinical chemistry and NMR spectroscopy. There was a high correlation for LDL-C (r = 0.86) and HDL-C (r = 0.88), but it was somewhat lower for triglycerides (r = 0.77). The TG correlation did not vary by fasting status (data not shown), and when using log transformed TG, the correlation was higher, at 0.91. Likewise, correlation coefficients for apolipoproteins A1 and B, creatinine, and albumin were high (0.87, 0.90, 0.98, and 0.80, respectively). The scatterplots for these 8 traits are presented in Online Figure 1.

The mean value for each metabolic measure, and the corresponding coefficient of variation from the 137 duplicate samples, are provided in Online Table 2. The coefficients of variation for duplicate measurements on the same samples had a median value of 5.0% (interquartile range: 2.7% to 6.7%).

### Comparisons of associations with mi of traits measured by nmr spectroscopy versus conventional clinical chemistry

When analyzed separately, the 8 biochemical traits measured by both conventional chemical chemistry and NMR spectroscopy assays indicated similar risk estimates for incident MI (Online Table 3). Positive associations with risk of MI were observed for total cholesterol, LDL-C, apolipoprotein B, TG, and creatinine, and inverse associations were observed for HDL-C, apolipoprotein A1, and albumin, for both assays.

### Associations with lipoprotein particle and lipid concentrations

**Figure 1:**
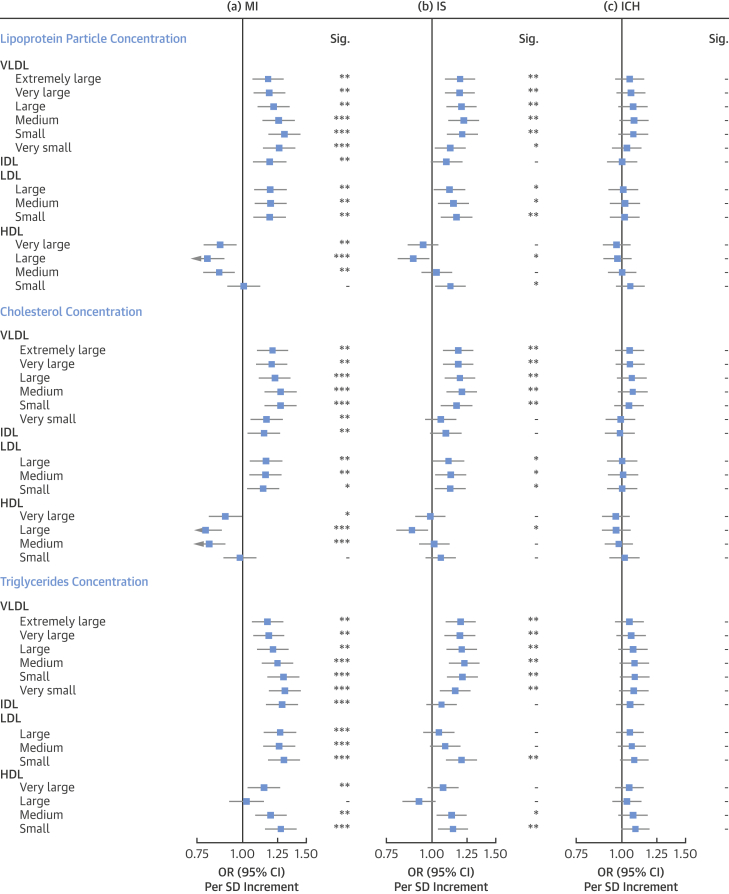
Adjusted OR (95% CI) of MI, IS, and ICH for Lipoprotein Particle Concentration, Cholesterol, and Triglycerides as Measured by Nuclear Magnetic Resonance Spectroscopy Estimates are odds ratios (ORs) per 1-SD higher metabolic marker. Models are adjusted for age, sex, fasting hours, region, smoking status, and educational attainment. Significance (Sig.): ∗∗∗p ≤ 0.0001, ∗∗p ≤ 0.01, ∗p ≤ 0.05, - p > 0.05 (false discovery rate [FDR]–adjusted p values). CI = confidence interval; HDL = high-density lipoprotein; ICH = intracerebral hemorrhage; IDL = intermediate-density lipoprotein; IS = ischemic stroke; LDL = low-density lipoprotein; MI = myocardial infarction; VLDL = very low-density lipoprotein.

As measured by NMR spectroscopy, there were positive associations with risk of MI with particle concentrations of very low-density lipoprotein (VLDL), intermediate-density lipoprotein (IDL), and LDL subclasses (after adjustment for multiple testing), with adjusted ORs per 1 SD increment ranging from 1.18 (95% CI: 1.07 to 1.29) for extremely large VLDL to 1.30 (95% CI: 1.19 to 1.44) for small VLDL. For HDL, very large (OR per SD increment: 0.87; 95% CI: 0.79 to 0.96), large (OR per SD increment: 0.80; 95% CI: 0.73 to 0.89), and medium (OR per SD increment: 0.86; 95% CI: 0.78 to 0.95) subclasses were inversely associated with risk of MI, but not small HDL (OR per SD increment: 1.01; 95% CI: 0.91 to 1.11). The estimates for cholesterol concentrations within individual lipoprotein subclasses were very similar to those of the corresponding lipoprotein particle concentrations (Figure 1).

For TG concentrations within lipoprotein subclasses, there were strong positive associations with risk of MI for TG in all apolipoprotein B–containing (VLDL, IDL, and LDL) lipoprotein particles. In contrast to cholesterol, TG in all HDL particles other than large HDL were positively associated with risk of MI.

For IS, the majority of VLDL particle subclasses were positively associated with disease risk; however, the associations were somewhat weaker for very small VLDL and IDL as compared with MI (e.g., OR per SD-higher IDL: 1.10 [95% CI: 1.00 to 1.20] for IS vs. 1.19 [95% CI: 1.08 to 1.31] for MI). Particle concentrations of large HDL were also inversely associated with IS (OR: 0.89; 95% CI: 0.81 to 0.98), whereas small HDL was positively associated with IS (OR: 1.12; 95% CI: 1.03 to 1.23). Likewise, the pattern of association with IS for cholesterol concentrations within lipoprotein particles was very similar to those for the concentrations of the lipoprotein particles themselves. TG concentrations within most VLDL and small LDL subclasses were also strongly positively associated with IS, but the associations of TG within very small VLDL, IDL, and large or medium LDL was weaker as compared with MI. As for MI, TG within medium and small HDL particles were positively associated with risk of IS.

In contrast, there were no clear associations of concentrations of any of the lipoprotein particles or their cholesterol or TG constituents with ICH (Figure 1).

### Associations with particle diameter, and cholesterol, TG, and apolipoprotein concentration

**Figure 2:**
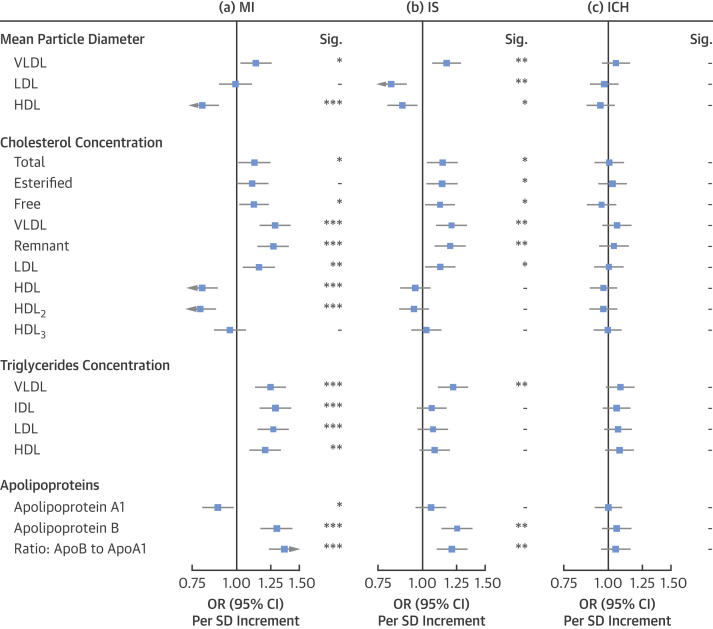
Adjusted OR (95% CI) of MI, IS, and ICH for Mean Particle Diameter, Cholesterol, Triglycerides, and Apolipoproteins as Measured by Nuclear Magnetic Resonance Spectroscopy Estimates are ORs per 1-SD higher metabolic marker. Models are adjusted for age, sex, fasting hours, region, smoking status, and educational attainment. Significance: ∗∗∗p ≤ 0.0001, ∗∗p ≤ 0.01, ∗p ≤ 0.05, - p > 0.05 (FDR-adjusted p values). ApoA1 = apolipoprotein A1; ApoB = apolipoprotein B; other abbreviations as in Figure 1.

VLDL diameter was positively associated with risk of MI (OR per SD: 1.13; 95% CI: 1.03 to 1.25), but LDL particle size had no association (OR per SD: 0.99; 95% CI: 0.90 to 1.10) and HDL particle size was inversely associated with MI (OR per SD: 0.80; 95% CI: 0.73 to 0.89) (Figure 2). For IS, there were positive associations with VLDL particle size (OR per SD: 1.17; 95% CI: 1.07 to 1.28) and inverse associations with HDL particle size (OR per SD: 0.88; 95% CI: 0.80 to 0.96). However, in contrast to MI, LDL particle size was inversely associated with IS (OR per SD 0.82; 95% CI: 0.74 to 0.90). Lipoprotein particle sizes were not associated with risk of ICH.

Remnant cholesterol concentrations (i.e., the cholesterol in VLDL and IDL particles) (15) were positively associated with increased risk of MI (OR per SD: 1.27; 95% CI: 1.15 to 1.39) and IS (OR per SD: 1.20; 95% CI: 1.09 to 1.32), but not ICH (OR per SD: 1.04; 95% CI: 0.95 to 1.13). Cholesterol within HDL_2_ (larger HDL particles) was strongly inversely associated with MI (OR per SD: 0.79; 95% CI: 0.72 to 0.87), but not with IS (OR per SD: 0.95; 95% CI: 0.86 to 1.04) or ICH (OR per SD: 0.97; 95% CI: 0.89 to 1.05), whereas cholesterol in HDL_3_ (smaller HDL particles) was not associated with any of the vascular disease endpoints.

TG in all measured major lipoprotein subclasses (apolipoprotein B–containing lipoproteins and HDL) were positively associated with higher risks of MI. In contrast, the positive association of TG with IS was limited to TG in VLDL. There was no association of TG with risk of ICH.

Concentrations of apolipoprotein A1 were inversely associated with risk of MI (OR per SD: 0.89; 95% CI: 0.81 to 0.98), but not with IS (OR per SD: 1.06; 95% CI: 0.96 to 1.16). In contrast, concentrations of apolipoprotein B and the ratio of apolipoprotein B to apolipoprotein A1 were both strongly positively associated with risk of MI (OR per SD: 1.30; 95% CI: 1.18 to 1.43; and 1.36; 95% CI: 1.24 to 1.50, respectively) and of IS (OR per SD: 1.25; 95% CI: 1.14 to 1.38, and 1.21; 95% CI: 1.10 to 1.33, respectively). None of the apolipoproteins, either individually or in ratios, was associated with risk of ICH.

### Associations with amino acids, fatty acids, ketone bodies, glycoprotein acetyls, and other traits

**Figure 3:**
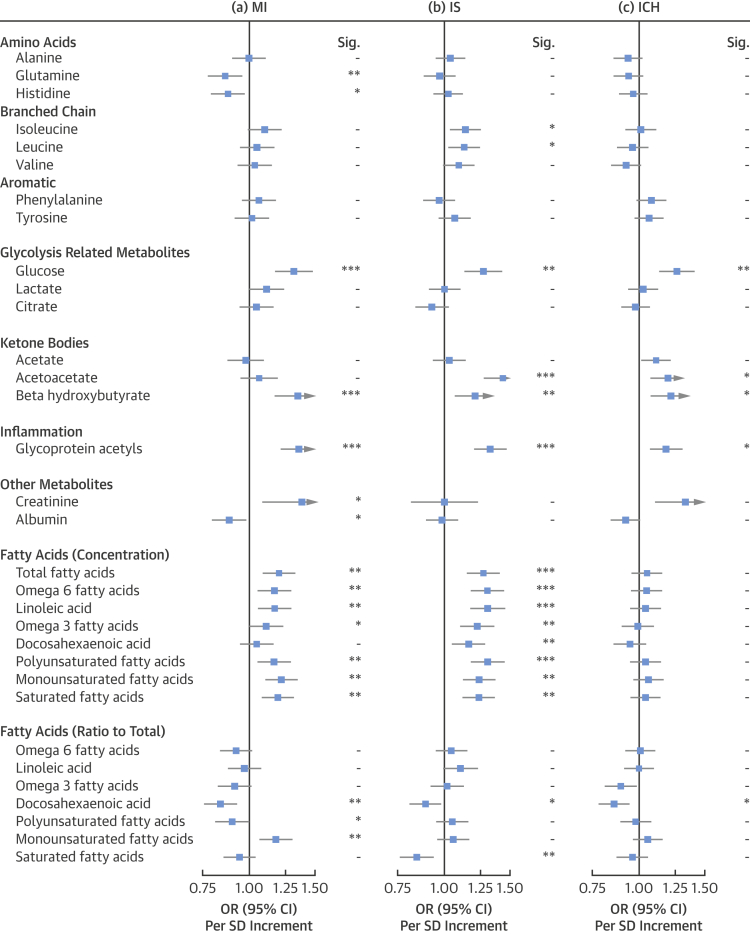
Adjusted OR (95% CI) of MI, IS, and ICH for Other Traits as Measured by NMR Spectroscopy Estimates are ORs per 1-SD higher metabolic marker. Models are adjusted for age, sex, fasting hours, region, smoking status, and educational attainment. Significance: ∗∗∗p ≤ 0.0001, ∗∗p ≤ 0.01, ∗p ≤ 0.05, - p > 0.05 (FDR-adjusted p values). Abbreviations as in Figure 1.

Among the amino acids measured, glutamine (OR per SD increment: 0.86; 95% CI: 0.78 to 0.95) and histidine (OR per SD increment: 0.88; 95% CI: 0.80 to 0.97) showed inverse associations with MI, and isoleucine and leucine showed modest positive associations with risk of IS. No amino acid showed an association with ICH (Figure 3). Glucose was positively associated with increased risk of MI (OR per SD increment: 1.32; 95% CI: 1.18 to 1.47), IS (OR per SD increment: 1.27; 95% CI: 1.14 to 1.42), and ICH (OR per SD increment: 1.26; 95% CI: 1.14 to 1.40). Among ketone bodies, β-hydroxybutyrate was associated with MI (OR per SD increment: 1.35; 95% CI: 1.18 to 1.54), IS (OR per SD increment: 1.21; 95% CI: 1.08 to 1.36), and ICH (OR per SD increment: 1.22; 95% CI: 1.08 to 1.36), and acetoacetate was strongly associated with IS (OR per SD increment: 1.44; 95% CI: 1.28 to 1.61) and ICH (OR per SD increment: 1.19; 95% CI: 1.08 to 1.32), but not with MI (OR per SD increment: 1.06; 95% CI: 0.95 to 1.18).

The inflammation marker, glycoprotein acetyls, was strongly associated with all 3 vascular diseases (for MI, OR per SD: 1.36; 95% CI: 1.22 to 1.50; for IS, OR per SD: 1.33; 95% CI: 1.21 to 1.46; and for ICH, OR per SD: 1.18; 95% CI: 1.08 to 1.30). There was a positive association of creatinine with MI (OR per SD: 1.38; 95% CI: 1.09 to 1.75) and nominally with ICH (OR per SD: 1.33; 95% CI: 1.11 to 1.59), but no association with IS (OR per SD: 1.00; 95% CI: 0.82 to 1.22).

Among the fatty acids, using the absolute concentrations, many were positively associated with risks of MI and IS. However, these findings were influenced by circulating TG levels, and adjustment for TG diminished all the associations for MI, whereas positive associations persisted for omega-6 fatty acids and polyunsaturated fatty acids for IS (Online Table 4). When reanalyzed as ratios, an inverse association of docosahexaenoic acid (an omega-3 fatty acid) as a proportion of total fatty acids was observed for MI, IS, and ICH. The ratio of monounsaturated fatty acids to total fatty acids showed a positive association with risk of MI, whereas the ratio of saturated fatty acids to total fatty acids showed an inverse association with risk of IS.

### Effects of additional adjustment for other cardiovascular risk factors

Further adjustment for SBP and BMI attenuated the association of lipoproteins, lipids and metabolites with risk of MI and IS (Online Figures 2 to 10). Similarly, additional adjustment for these factors also diminished the associations of several traits that were associated with ICH, particularly glycoprotein acetyls and creatinine. Interestingly, after adjustment for SBP, some traits such as albumin showed stronger inverse associations with risk of MI (Online Figure 4) and an inverse association of albumin with ICH emerged (Online Figure 10). Adjustment for SBP and BMI had no material impact on the generally neutral associations of lipoproteins and lipid traits with risk of ICH.

### Comparison of overall risk associations for MI, IS, and ICH

**Figure 4:**
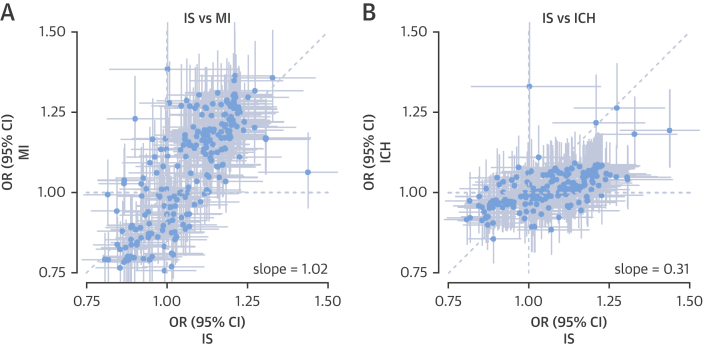
Comparison of Adjusted OR (95% CI) of 225 Metabolic Measures as Measured by Nuclear Magnetic Resonance Spectroscopy Comparison between IS and **(A)** MI and **(B)** ICH. Estimates are per 1-SD higher metabolic marker. Model adjustment as in Figure 1. Abbreviations as in Figure 1.

In analyses of all 225 traits measured by NMR spectroscopy with disease risks, there was general concordance of the patterns of association between MI and IS (i.e., metabolic measures that associated with MI tended to associate with IS; regression slope = 1.02) (Figure 4A). In contrast, there was less of a concordance of the associations of traits with risk of IS and ICH, with traits showing, in general, much weaker associations with ICH (regression slope = 0.31), with a few notable exceptions (Figure 4B).

### Discriminatory ability

Addition of 18 principal components that explained 95% of the variance of the 225 metabolic markers into a logistic regression model with established risk factors yielded very small, but statistically significant, increases in the area under the receiver-operating characteristic curve (increases ranged from 0.008 to 0.013) for the 3 vascular endpoints (Online Table 5).

## Discussion

This study provides a comprehensive examination of the associations of lipoprotein particles, lipid constituents, and multiple circulating metabolites with risks of incident MI, IS, and ICH. The results show that MI and IS have broadly similar strengths of association with concentrations of lipoprotein and lipid constituents, but for ICH the associations were substantially weaker (Central Illustration). In contrast, certain non–lipid-related metabolites, such as glycoprotein acetyls and glucose, showed similar strengths of association for all 3 subtypes of CVD.

**Central Illustration:**
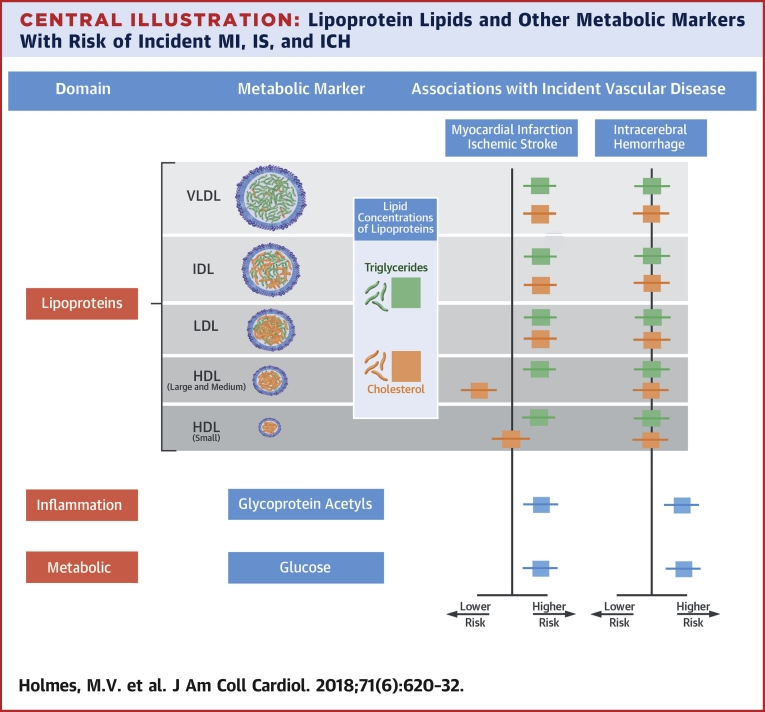
Lipoprotein Lipids and Other Metabolic Markers With Risk of Incident MI, IS, and ICH We assessed the associations of metabolic markers measured by nuclear magnetic resonance (NMR) spectroscopy with risk of incident myocardial infarction (MI), ischemic stroke (IS), and intracerebral hemorrhage (ICH). The study demonstrated that lipoprotein subclasses and their lipid constituents shared associations with both risk of MI and IS, but not with risk of ICH. For MI and IS, cholesterol **(orange squares and horizontal lines)** and triglycerides **(green squares and horizontal lines)** in apolipoprotein B–containing lipoproteins (very low-density lipoprotein [VLDL], intermediate-density lipoprotein [IDL], and low-density lipoprotein [LDL]) were positively associated with risk of both diseases. In contrast, cholesterol in large and medium high-density lipoprotein (HDL) particles was inversely associated with risk of MI and IS, whereas triglycerides in HDL particles were positively associated with disease risk. Neither lipoproteins nor lipid constituents showed associations with risk of ICH. In contrast, glucose and the inflammation marker glycoprotein acetyls were both associated with higher risks of all 3 diseases. Thus, although lipids and lipoproteins appear to be less relevant to ICH than MI or IS, the association of some metabolites with MI, IS, and ICH indicates shared pathways for all 3 subtypes of vascular disease.

Concentrations of the apolipoprotein B–containing lipoproteins and their cholesterol concentrations showed positive associations with risks of MI, which is consistent with the known causal effects of LDL-C on risk of MI from genetic studies 16, 17, 18, and from randomized controlled trials of LDL-C lowering therapies 3, 4, 19. The slightly weaker association of these lipid-related traits with risk of IS as compared with MI is also consistent with previous studies of conventionally measured lipids (1), and of metabolomics in individuals with prior vascular disease or diabetes (20).

Importantly, the TG within all apolipoprotein B and most HDL particles were associated with higher risk of MI and, to a slightly lesser extent, of IS. The positive association of TG in small and medium HDL lipoproteins differed from the inverse association of cholesterol in large and medium HDL lipoproteins with MI (and IS), which raises the hypothesis that TG and cholesterol in HDL particles have opposing relationships with risk of MI or IS. This is in contrast to the consistent associations of TG and cholesterol in apolipoprotein B–containing lipoproteins with risk of MI or IS.

Consistent with previous observational studies 6, 20, we identified inverse associations of medium and large HDL particles and cholesterol concentrations within these lipoproteins with risk of MI. Although this could be explained by residual confounding or reverse causality, we note that most genetic investigations of HDL in CHD to date 16, 17, 21, 22 have used genetic variants associated with HDL-C overall, a composite measure of the sum of cholesterol concentrations across HDL lipoproteins. The collective findings of the present and recent studies 6, 23 indicate that the inverse observational associations of cholesterol in HDL particles are limited to large and medium subclasses. As such, it is plausible that inclusion of genetic variants in previous studies that associate disease risk with heterogeneous HDL particles (24) could mask a potential “true” protective effect constrained to medium and large HDL; however, medium and large HDL particles make a substantial contribution to most measures of circulating HDL-C.

One of the most striking findings from the present study is the absence of association of any lipoprotein, apolipoprotein, or lipid constituent with risk of ICH. Although the observed associations differ from previous studies that reported inverse associations of LDL-C with risk of ICH (5), our study suggests ICH is possibly less driven by atherosclerosis and more so by hypertension and frailty of blood vessels. However, a modest correlation between the directions of association for IS and ICH (Figure 4) indicates it is possible that the individual null findings for ICH reflect a lack of statistical power in the present study to detect modest, but potentially real, associations of these measures with ICH.

Several other non–lipid-related metabolic markers did show strong associations with ICH. For example, glucose traits and the inflammation marker glycoprotein acetyls were similarly associated with risk of ICH as to risk of MI and IS. Glycoprotein acetyls are a marker of glycosylated proteins, acute-phase reactants present in very small quantities in the blood 25, 26, 27, 28. They are moderately correlated with C-reactive protein (29) and recent studies 6, 30, 31 reported elevated CVD risks associated with higher concentrations of glycoprotein acetyls, as in the present study. Other studies have also shown glycoprotein acetyls to associate with hypertension, plasma glucose (29), and incident type 2 diabetes (32). To our knowledge, the present study is the first to quantify its association with risk of incident ICH and demonstrate consistent associations of glycoprotein acetyls across MI, IS, and ICH.

The present study adds new knowledge to a recent study in Europeans (6) by demonstrating consistent associations of cholesterol and TG when present in apolipoprotein B–containing lipoproteins, yet differential associations of cholesterol and TG when present in HDL lipoproteins. The present study did not replicate the association of phenylalanine with risk of ischemic CVD (6), potentially reflecting effect modification by an environmental exposure such as dietary factors (11). This study also adds new information on the associations of these metabolic markers with ICH.

Our study has several strengths. The availability of 8 traits measured by both NMR spectroscopy and accredited clinical chemistry assays, and 137 duplicate samples of NMR metabolomics, provides tests of internal validity. This is one of the largest studies of the relationship of blood-based metabolic markers and risk of incident vascular disease subtypes. The absence of drug treatment by statins or other lipid-modifying drugs at the time of blood sampling, and the incident nature of vascular cases, reduces sources of potential bias. Analysis of 3 different diseases (MI, IS, and ICH) in the same study facilitated direct comparison of risk estimates of metabolic markers with these diseases.

### Study limitations

The lack of associations of lipoproteins and lipids with risk of ICH may reflect low statistical power. The low mean levels of LDL-C in the present study could challenge extrapolation of our findings to Western populations; however, the INTERHEART global study of risk factors for acute myocardial infarction (33) found similar associations of lipoproteins with MI irrespective of geographical region. The NMR spectroscopy assay did not quantify lipoprotein(a), apolipoprotein CIII, and HDL functionality, which would be interesting additions to the present study. There is substantial collinearity within the metabolomics data, with multiple lipid measures representing subsets of strongly correlated lipid factions. This can obscure the interpretation of individual trait associations, making it difficult to clarify which lipoprotein characteristic (concentration, particle size, lipid constituent) accounts for disease associations. Nevertheless, this does not detract from the insights evident where there were clear differences in the patterns of associations (e.g., when comparing small and large HDL particles, or when comparing TG and cholesterol in HDL particles).

## Conclusions

Lipoproteins and lipids show generally consistent associations with risks of MI and IS. The present study demonstrated opposing associations of cholesterol and TG in HDL particles with risk of MI and IS. Although lipids and lipoproteins may play a less important role in the development of ICH as compared with MI or IS, inflammation may play a role in MI, IS, and ICH. Large-scale studies using genetic approaches are needed to clarify the causal roles of individual lipoproteins, lipid constituents, and other metabolic markers for subtypes of stroke and MI.

Perspectives **COMPETENCY IN PATIENT CARE AND PROCEDURAL SKILLS:** Various lipoprotein subclasses and lipid constituents are associated with similar risks of incident MI and IS, but not with hemorrhagic stroke. In contrast, markers of inflammation and glucose are related to the incidence of these disparate events.

**TRANSLATIONAL OUTLOOK:** Detailed lipoprotein and lipid profiling can clarify the relationships of lipids with risk of vascular diseases. This is useful to clarify which lipoproteins and lipids are important for disease risk, and can shed light on disease etiology.
