# Supplementary material for: Lipids, Lipoproteins, and Metabolites and Risk of Myocardial Infarction and Stroke
Source: J Am Coll Cardiol. 2018 Feb 13;71(6):620–32. doi: 10.1016/j.jacc.2017.12.006 (PMC5811927; doi:10.1016/j.jacc.2017.12.006)

**Supplementary Online Content**

Holmes MV, Millwood IY, Kartsonaki C et al. Lipids, lipoproteins and metabolites and risk of myocardial infarction and stroke

Table of Contents

[Online Table 1. Correlation coefficients of eight traits measured by clinical chemistry and NMR spectroscopy 2](#_Toc502940287)

[Online Table 2. Mean concentrations in control subjects and adjusted ORs (95%CI) of myocardial infarction, ischemic stroke and intracerebral hemorrhage for each metabolic marker 3](#_Toc502940288)

[Online Table 3. Adjusted ORs (95% CI) of myocardial infarction for traits measured by clinical chemistry and NMR spectroscopy 13](#_Toc502940289)

[Online Table 4. Adjusted ORs (95% CI) of myocardial infarction and ischemic stroke for fatty acids additionally adjusted for triglycerides 14](#_Toc502940290)

[Online Table 5. Discriminatory ability 15](#_Toc502940291)

[Online Figure 1. Scatterplots of 8 traits measured by both clinical chemistry and NMR spectroscopy 16](#_Toc502940292)

[Online Figure 2. Adjusted ORs (95% CI) of myocardial infarction for lipoprotein particle concentration, cholesterol and triglycerides, with additional adjustment for SBP and BMI 17](#_Toc502940293)

[Online Figure 3. Adjusted ORs (95% CI) of myocardial infarction for particle diameter, cholesterol and triglycerides, with additional adjustment for SBP and BMI 18](#_Toc502940294)

[Online Figure 4. Adjusted ORs (95% CI) of myocardial infarction for other traits, with additional adjustment for SBP and BMI 19](#_Toc502940295)

[Online Figure 5. Adjusted ORs (95% CI) of ischemic stroke for lipoprotein particle concentration, cholesterol and triglycerides, with additional adjustment for SBP and BMI 20](#_Toc502940296)

[Online Figure 6. Adjusted ORs (95% CI) of ischemic stroke for particle diameter, cholesterol and triglycerides, with additional adjustment for SBP and BMI 21](#_Toc502940297)

[Online Figure 7. Adjusted ORs (95% CI) of ischemic stroke for other traits, with additional adjustment for SBP and BMI 22](#_Toc502940298)

[Online Figure 8. Adjusted ORs (95% CI) of intracerebral hemorrhage for lipoprotein particle concentration, cholesterol and triglycerides, with additional adjustment for SBP and BMI 23](#_Toc502940299)

[Online Figure 9. Adjusted ORs (95% CI) of intracerebral hemorrhage for particle diameter, cholesterol and triglycerides, with additional adjustment for SBP and BMI 24](#_Toc502940300)

[Online Figure 10. Adjusted ORs (95% CI) of intracerebral hemorrhage for other traits, with additional adjustment for SBP and BMI 25](#_Toc502940301)

# Online Table 1. Correlation coefficients of eight traits measured by clinical chemistry and NMR spectroscopy

| **Trait** | **N** | **Mean (standard deviation)** | | **Correlation**  **Coefficient*** |
| --- | --- | --- | --- | --- |
|  |  | **Clinical Chemistry** | **NMR spectroscopy** |  |
| **Lipids (mg/L)** | | | | |
| Total cholesterol | 4661 | 177.6 (38.6) | 135.1 (23.2) | 0.84 |
| LDL-cholesterol † | 4661 | 88.8 (27.0) | 42.5 (15.4) | 0.86 |
| HDL-cholesterol | 4661 | 46.3 (11.6) | 50.2 (7.7) | 0.88 |
| Triglycerides | 4661 | 185.8 (150.4) | 115.0 (44.2) | 0.77‡ |
| **Lipoproteins (mg/dL)** | | | | |
| Apolipoprotein A-1 | 4661 | 126.6 (18.2) | 140.5 (12.2) | 0.87 |
| Apolipoprotein B | 4659 | 82.8 (21.1) | 73.5 (13.7) | 0.90 |
| **Renal function and plasma proteins** | | | | |
| Creatinine (µmol/L) | 4635 | 62.41 (31.21) | 63.5 (32.3) | 0.98 |
| Albumin § | 4662 | 42.97 (2.72) | 0.09 (0.003) | 0.80 |

**Legend**

*Correlations are Pearson’s correlation coefficients
† For clinical chemistry assay, LDL-cholesterol was directly measured (and was not estimated using Friedewald)

‡ For triglycerides, the Spearman’s rank based coefficient was 0.93 and for log(triglycerides) the Pearson’s correlation coefficient was 0.91.

§ Units for albumin are signal area for NMR and g/L for clinical biochemistry
To convert HDL-cholesterol and LDL-cholesterol to mmol/L, multiply by 0.0259; to convert triglycerides to mmol/L, multiply by 0.0113. To convert apolipoprotein A-1 to mmol/L, multiply by 0.0004; to convert apolipoprotein B to mmol/l, multiply by 0.00001949317738791423.

# Online Table 2. Mean concentrations in control subjects and adjusted ORs (95%CI) of myocardial infarction, ischemic stroke and intracerebral hemorrhage for each metabolic marker

| **Metabolic measure (units)** | **N** | **Mean (SD) in control subjects** | **CV (%)** | **Adjusted ORs (95% CI) per 1 SD higher metabolite** | | |
| --- | --- | --- | --- | --- | --- | --- |
|  |  |  |  | **Myocardial infarction** | **Ischemic stroke** | **Intracerebral hemorrhage** |
| Acetate (mmol/l) | 1466 | 0.05 (0.067) | 8.29 | 0.98 (0.88, 1.09) | 1.03 (0.94, 1.13) | 1.11 (1.02, 1.21) |
| Acetoacetate (mmol/l) | 1466 | 0.023 (0.011) | 13.95 | 1.06 (0.95, 1.18) | 1.44 (1.28, 1.61) | 1.19 (1.08, 1.32) |
| Alanine (mmol/l) | 1466 | 0.4 (0.061) | 4.69 | 1.00 (0.91, 1.10) | 1.04 (0.95, 1.13) | 0.93 (0.86, 1.01) |
| Albumin (signal area) | 1466 | 0.085 (0.0028) | 1.58 | 0.88 (0.80, 0.98) | 0.99 (0.90, 1.08) | 0.92 (0.85, 1.00) |
| Apolipoprotein A-I (g/l) | 1466 | 1.4 (0.12) | 1.71 | 0.89 (0.81, 0.98) | 1.06 (0.96, 1.16) | 1.00 (0.92, 1.09) |
| Apolipoprotein B (g/l) | 1465 | 0.72 (0.13) | 4.33 | 1.30 (1.18, 1.43) | 1.25 (1.14, 1.38) | 1.05 (0.96, 1.15) |
| Cholesterol esters in chylomicrons and extremely large VLDL (mmol/l) | 1466 | 0.0027 (0.0021) | 12.84 | 1.24 (1.13, 1.36) | 1.18 (1.08, 1.29) | 1.05 (0.96, 1.15) |
| Cholesterol esters in IDL (mmol/l) | 1466 | 0.34 (0.086) | 7.60 | 1.17 (1.06, 1.29) | 1.12 (1.02, 1.23) | 0.99 (0.91, 1.09) |
| Cholesterol esters in large HDL (mmol/l) | 1466 | 0.26 (0.084) | 4.12 | 0.79 (0.72, 0.87) | 0.88 (0.80, 0.97) | 0.97 (0.89, 1.05) |
| Cholesterol esters in large LDL (mmol/l) | 1466 | 0.4 (0.12) | 6.92 | 1.18 (1.07, 1.30) | 1.13 (1.03, 1.24) | 1.01 (0.92, 1.10) |
| Cholesterol esters in large VLDL (mmol/l) | 1466 | 0.034 (0.02) | 9.04 | 1.24 (1.13, 1.37) | 1.18 (1.08, 1.29) | 1.06 (0.97, 1.16) |
| Cholesterol esters in medium HDL (mmol/l) | 1466 | 0.31 (0.051) | 3.72 | 0.81 (0.73, 0.89) | 1.02 (0.93, 1.12) | 0.98 (0.90, 1.07) |
| Cholesterol esters in medium LDL (mmol/l) | 1466 | 0.21 (0.087) | 6.95 | 1.15 (1.05, 1.27) | 1.12 (1.02, 1.23) | 1.01 (0.92, 1.11) |
| Cholesterol esters in medium VLDL (mmol/l) | 1466 | 0.076 (0.033) | 9.15 | 1.28 (1.16, 1.40) | 1.19 (1.09, 1.31) | 1.06 (0.97, 1.16) |
| Cholesterol esters in small HDL (mmol/l) | 1466 | 0.28 (0.051) | 5.09 | 1.00 (0.91, 1.10) | 1.04 (0.95, 1.14) | 1.01 (0.92, 1.11) |
| Cholesterol esters in small LDL (mmol/l) | 1466 | 0.14 (0.054) | 5.88 | 1.14 (1.03, 1.25) | 1.12 (1.02, 1.22) | 1.00 (0.92, 1.10) |
| Cholesterol esters in small VLDL (mmol/l) | 1466 | 0.1 (0.032) | 8.68 | 1.24 (1.13, 1.36) | 1.14 (1.04, 1.25) | 1.03 (0.94, 1.13) |
| Cholesterol esters in very large HDL (mmol/l) | 1466 | 0.17 (0.041) | 6.64 | 0.91 (0.82, 1.00) | 1.00 (0.92, 1.10) | 0.97 (0.89, 1.05) |
| Cholesterol esters in very large VLDL (mmol/l) | 1466 | 0.0085 (0.0062) | 10.87 | 1.21 (1.10, 1.33) | 1.17 (1.07, 1.28) | 1.05 (0.97, 1.15) |
| Cholesterol esters in very small VLDL (mmol/l) | 1466 | 0.13 (0.029) | 7.73 | 1.17 (1.06, 1.29) | 1.06 (0.96, 1.16) | 0.99 (0.91, 1.09) |
| Cholesterol esters to total lipids ratio in chylomicrons and extremely large VLDL (%) | 1386 | 9.7 (3.6) | 41.63 | 1.16 (1.05, 1.29) | 0.96 (0.86, 1.06) | 1.02 (0.94, 1.12) |
| Cholesterol esters to total lipids ratio in IDL (%) | 1464 | 44 (2.2) | 2.17 | 1.03 (0.94, 1.14) | 1.18 (1.08, 1.30) | 0.97 (0.90, 1.06) |
| Cholesterol esters to total lipids ratio in large HDL (%) | 1465 | 37 (1.7) | 0.85 | 0.79 (0.72, 0.87) | 0.87 (0.80, 0.96) | 0.92 (0.85, 1.01) |
| Cholesterol esters to total lipids ratio in large LDL (%) | 1463 | 45 (3.7) | 1.58 | 1.08 (0.98, 1.19) | 1.16 (1.05, 1.27) | 1.02 (0.94, 1.11) |
| Cholesterol esters to total lipids ratio in large VLDL (%) | 1438 | 12 (1.7) | 4.71 | 1.03 (0.93, 1.14) | 0.87 (0.79, 0.95) | 0.92 (0.85, 1.01) |
| Cholesterol esters to total lipids ratio in medium HDL (%) | 1465 | 40 (2.1) | 0.95 | 0.77 (0.70, 0.85) | 1.01 (0.92, 1.11) | 0.94 (0.87, 1.02) |
| Cholesterol esters to total lipids ratio in medium LDL (%) | 1450 | 42 (7.2) | 1.90 | 1.00 (0.91, 1.10) | 1.04 (0.95, 1.15) | 1.00 (0.92, 1.09) |
| Cholesterol esters to total lipids ratio in medium VLDL (%) | 1465 | 14 (2.4) | 6.44 | 1.01 (0.91, 1.12) | 0.94 (0.85, 1.03) | 0.97 (0.89, 1.06) |
| Cholesterol esters to total lipids ratio in small HDL (%) | 1464 | 29 (4.5) | 2.77 | 1.01 (0.92, 1.11) | 0.98 (0.90, 1.08) | 0.99 (0.91, 1.08) |
| Cholesterol esters to total lipids ratio in small LDL (%) | 1449 | 40 (7.1) | 1.67 | 0.99 (0.90, 1.09) | 1.02 (0.93, 1.12) | 1.00 (0.91, 1.09) |
| Cholesterol esters to total lipids ratio in small VLDL (%) | 1464 | 20 (4) | 5.76 | 0.94 (0.85, 1.04) | 0.94 (0.86, 1.03) | 0.96 (0.88, 1.05) |
| Cholesterol esters to total lipids ratio in very large HDL (%) | 1465 | 37 (3.7) | 3.17 | 1.17 (1.06, 1.29) | 1.20 (1.09, 1.31) | 1.01 (0.92, 1.10) |
| Cholesterol esters to total lipids ratio in very large VLDL (%) | 1359 | 13 (2.4) | 5.07 | 1.04 (0.94, 1.15) | 0.87 (0.79, 0.96) | 1.01 (0.93, 1.10) |
| Cholesterol esters to total lipids ratio in very small VLDL (%) | 1465 | 34 (2.7) | 4.33 | 0.85 (0.77, 0.94) | 0.89 (0.81, 0.98) | 0.91 (0.83, 0.99) |
| Citrate (mmol/l) | 1466 | 0.13 (0.02) | 5.92 | 1.04 (0.95, 1.15) | 0.93 (0.84, 1.02) | 0.98 (0.90, 1.06) |
| Concentration of chylomicrons and extremely large VLDL particles (mol/l) | 1466 | 1.3e-10 (1.1e-10) | 13.19 | 1.18 (1.07, 1.29) | 1.20 (1.09, 1.31) | 1.05 (0.97, 1.15) |
| Concentration of IDL particles (mol/l) | 1466 | 7.6e-08 (1.7e-08) | 5.75 | 1.19 (1.08, 1.31) | 1.10 (1.00, 1.20) | 1.00 (0.92, 1.10) |
| Concentration of large HDL particles (mol/l) | 1466 | 1.1e-06 (3.2e-07) | 3.62 | 0.80 (0.73, 0.89) | 0.89 (0.81, 0.98) | 0.97 (0.90, 1.06) |
| Concentration of large LDL particles (mol/l) | 1466 | 1.2e-07 (3e-08) | 5.70 | 1.19 (1.08, 1.32) | 1.12 (1.02, 1.23) | 1.01 (0.93, 1.11) |
| Concentration of large VLDL particles (mol/l) | 1466 | 4.9e-09 (3.1e-09) | 9.34 | 1.22 (1.11, 1.34) | 1.21 (1.10, 1.32) | 1.07 (0.99, 1.17) |
| Concentration of medium HDL particles (mol/l) | 1466 | 1.8e-06 (2.7e-07) | 3.22 | 0.86 (0.78, 0.95) | 1.03 (0.94, 1.13) | 1.00 (0.92, 1.09) |
| Concentration of medium LDL particles (mol/l) | 1466 | 9.7e-08 (2.8e-08) | 5.29 | 1.20 (1.09, 1.32) | 1.15 (1.04, 1.26) | 1.02 (0.94, 1.12) |
| Concentration of medium VLDL particles (mol/l) | 1466 | 1.7e-08 (7.5e-09) | 7.15 | 1.26 (1.14, 1.38) | 1.22 (1.12, 1.34) | 1.08 (0.99, 1.18) |
| Concentration of small HDL particles (mol/l) | 1466 | 4.5e-06 (4e-07) | 2.59 | 1.01 (0.91, 1.11) | 1.12 (1.03, 1.23) | 1.06 (0.97, 1.15) |
| Concentration of small LDL particles (mol/l) | 1466 | 1.2e-07 (3.1e-08) | 5.72 | 1.19 (1.08, 1.31) | 1.17 (1.06, 1.28) | 1.02 (0.93, 1.11) |
| Concentration of small VLDL particles (mol/l) | 1466 | 2.7e-08 (7.6e-09) | 5.18 | 1.30 (1.19, 1.44) | 1.21 (1.10, 1.33) | 1.08 (0.99, 1.18) |
| Concentration of very large HDL particles (mol/l) | 1466 | 4.5e-07 (1.3e-07) | 4.72 | 0.87 (0.79, 0.96) | 0.94 (0.86, 1.03) | 0.97 (0.89, 1.05) |
| Concentration of very large VLDL particles (mol/l) | 1466 | 7.4e-10 (6e-10) | 11.40 | 1.19 (1.08, 1.30) | 1.19 (1.09, 1.30) | 1.06 (0.97, 1.16) |
| Concentration of very small VLDL particles (mol/l) | 1466 | 3.1e-08 (6.3e-09) | 4.55 | 1.26 (1.15, 1.39) | 1.12 (1.02, 1.23) | 1.03 (0.95, 1.13) |
| Creatinine (mmol/l) | 1455 | 0.051 (0.013) | 5.67 | 1.38 (1.09, 1.75) | 1.00 (0.82, 1.22) | 1.33 (1.11, 1.59) |
| Esterified cholesterol (mmol/l) | 1465 | 2 (0.43) | 5.19 | 1.11 (1.00, 1.22) | 1.14 (1.03, 1.25) | 1.03 (0.94, 1.12) |
| Estimated degree of unsaturation | 1462 | 1.1 (0.054) | 1.39 | 0.90 (0.81, 0.99) | 1.07 (0.97, 1.19) | 0.88 (0.81, 0.97) |
| Free cholesterol (mmol/l) | 1465 | 0.94 (0.2) | 5.24 | 1.12 (1.02, 1.22) | 1.12 (1.02, 1.23) | 0.96 (0.88, 1.05) |
| Free cholesterol in chylomicrons and extremely large VLDL (mmol/l) | 1466 | 0.002 (0.0019) | 13.48 | 1.17 (1.07, 1.28) | 1.18 (1.08, 1.29) | 1.06 (0.97, 1.15) |
| Free cholesterol in IDL (mmol/l) | 1466 | 0.13 (0.037) | 5.68 | 1.08 (0.98, 1.19) | 1.03 (0.94, 1.13) | 0.98 (0.90, 1.07) |
| Free cholesterol in large HDL (mmol/l) | 1466 | 0.073 (0.026) | 4.43 | 0.79 (0.71, 0.87) | 0.88 (0.80, 0.96) | 0.96 (0.88, 1.05) |
| Free cholesterol in large LDL (mmol/l) | 1466 | 0.17 (0.041) | 5.45 | 1.11 (1.01, 1.22) | 1.06 (0.97, 1.16) | 0.99 (0.90, 1.08) |
| Free cholesterol in large VLDL (mmol/l) | 1466 | 0.026 (0.021) | 11.66 | 1.21 (1.10, 1.33) | 1.20 (1.10, 1.31) | 1.07 (0.98, 1.17) |
| Free cholesterol in medium HDL (mmol/l) | 1466 | 0.064 (0.013) | 4.58 | 0.83 (0.76, 0.92) | 0.99 (0.91, 1.09) | 0.99 (0.91, 1.08) |
| Free cholesterol in medium LDL (mmol/l) | 1466 | 0.1 (0.021) | 4.57 | 1.16 (1.06, 1.28) | 1.13 (1.03, 1.24) | 1.01 (0.93, 1.10) |
| Free cholesterol in medium VLDL (mmol/l) | 1466 | 0.062 (0.031) | 8.20 | 1.26 (1.15, 1.39) | 1.22 (1.11, 1.33) | 1.08 (0.99, 1.18) |
| Free cholesterol in small HDL (mmol/l) | 1466 | 0.1 (0.011) | 2.63 | 0.93 (0.85, 1.03) | 1.07 (0.98, 1.17) | 1.03 (0.95, 1.12) |
| Free cholesterol in small LDL (mmol/l) | 1466 | 0.061 (0.013) | 5.87 | 1.15 (1.04, 1.27) | 1.14 (1.04, 1.26) | 1.00 (0.92, 1.09) |
| Free cholesterol in small VLDL (mmol/l) | 1466 | 0.07 (0.019) | 5.13 | 1.29 (1.17, 1.41) | 1.19 (1.08, 1.30) | 1.07 (0.98, 1.17) |
| Free cholesterol in very large HDL (mmol/l) | 1466 | 0.057 (0.018) | 6.69 | 0.87 (0.79, 0.96) | 0.95 (0.87, 1.04) | 0.95 (0.88, 1.04) |
| Free cholesterol in very large VLDL (mmol/l) | 1466 | 0.0057 (0.0055) | 14.44 | 1.19 (1.09, 1.31) | 1.19 (1.09, 1.30) | 1.06 (0.97, 1.15) |
| Free cholesterol in very small VLDL (mmol/l) | 1466 | 0.059 (0.014) | 4.49 | 1.15 (1.04, 1.26) | 1.05 (0.96, 1.15) | 1.00 (0.91, 1.09) |
| Free cholesterol to total lipids ratio in chylomicrons and extremely large VLDL (%) | 1386 | 6.4 (1.7) | 7.45 | 1.06 (0.97, 1.16) | 1.07 (0.98, 1.18) | 1.04 (0.95, 1.13) |
| Free cholesterol to total lipids ratio in IDL (%) | 1464 | 17 (1.9) | 1.00 | 0.88 (0.81, 0.97) | 0.90 (0.83, 0.99) | 0.96 (0.89, 1.05) |
| Free cholesterol to total lipids ratio in large HDL (%) | 1465 | 10 (0.99) | 2.69 | 0.78 (0.71, 0.86) | 0.87 (0.79, 0.95) | 0.92 (0.85, 1.00) |
| Free cholesterol to total lipids ratio in large LDL (%) | 1463 | 20 (1.2) | 0.86 | 0.79 (0.72, 0.87) | 0.81 (0.74, 0.89) | 0.92 (0.84, 1.00) |
| Free cholesterol to total lipids ratio in large VLDL (%) | 1438 | 8.4 (2) | 3.15 | 1.13 (1.03, 1.25) | 1.17 (1.07, 1.28) | 1.04 (0.95, 1.13) |
| Free cholesterol to total lipids ratio in medium HDL (%) | 1465 | 8.4 (0.58) | 1.34 | 0.80 (0.72, 0.87) | 0.93 (0.85, 1.02) | 0.96 (0.89, 1.05) |
| Free cholesterol to total lipids ratio in medium LDL (%) | 1450 | 21 (2.1) | 1.31 | 0.89 (0.81, 0.98) | 0.89 (0.81, 0.97) | 0.95 (0.87, 1.04) |
| Free cholesterol to total lipids ratio in medium VLDL (%) | 1465 | 11 (0.88) | 1.02 | 1.19 (1.08, 1.32) | 1.13 (1.04, 1.23) | 1.07 (0.99, 1.17) |
| Free cholesterol to total lipids ratio in small HDL (%) | 1464 | 10 (0.51) | 1.13 | 0.86 (0.78, 0.95) | 0.94 (0.86, 1.04) | 0.97 (0.89, 1.06) |
| Free cholesterol to total lipids ratio in small LDL (%) | 1449 | 19 (1.5) | 1.28 | 0.88 (0.80, 0.97) | 0.86 (0.79, 0.95) | 0.94 (0.86, 1.03) |
| Free cholesterol to total lipids ratio in small VLDL (%) | 1464 | 14 (0.56) | 0.95 | 0.90 (0.81, 0.99) | 0.89 (0.82, 0.98) | 0.97 (0.89, 1.05) |
| Free cholesterol to total lipids ratio in very large HDL (%) | 1465 | 13 (0.69) | 3.49 | 0.95 (0.87, 1.05) | 1.01 (0.92, 1.11) | 0.89 (0.82, 0.98) |
| Free cholesterol to total lipids ratio in very large VLDL (%) | 1359 | 7.1 (2) | 8.41 | 1.12 (1.02, 1.25) | 1.16 (1.05, 1.27) | 0.97 (0.89, 1.06) |
| Free cholesterol to total lipids ratio in very small VLDL (%) | 1465 | 15 (1.4) | 1.72 | 0.89 (0.81, 0.98) | 0.92 (0.84, 1.00) | 0.95 (0.88, 1.04) |
| Glucose (mmol/l) | 1461 | 3.9 (1.7) | 2.24 | 1.32 (1.18, 1.47) | 1.27 (1.14, 1.42) | 1.26 (1.14, 1.40) |
| Glutamine (mmol/l) | 1462 | 0.47 (0.064) | 6.32 | 0.86 (0.78, 0.95) | 0.97 (0.89, 1.07) | 0.94 (0.86, 1.02) |
| Glycoprotein acetyls mainly a1 acid glycoprotein (mmol/l) | 1466 | 1.3 (0.2) | 4.36 | 1.36 (1.22, 1.50) | 1.33 (1.21, 1.46) | 1.18 (1.08, 1.30) |
| Histidine (mmol/l) | 1462 | 0.063 (0.0064) | 7.84 | 0.88 (0.80, 0.97) | 1.02 (0.94, 1.12) | 0.96 (0.89, 1.05) |
| Isoleucine (mmol/l) | 1466 | 0.06 (0.018) | 8.11 | 1.10 (1.00, 1.21) | 1.14 (1.04, 1.25) | 1.01 (0.92, 1.10) |
| Lactate (mmol/l) | 1466 | 2.5 (0.79) | 5.19 | 1.11 (1.01, 1.23) | 1.00 (0.92, 1.10) | 1.02 (0.94, 1.12) |
| Leucine (mmol/l) | 1466 | 0.076 (0.019) | 5.50 | 1.05 (0.95, 1.16) | 1.13 (1.03, 1.24) | 0.96 (0.88, 1.05) |
| Mean diameter for HDL particles (nm) | 1466 | 10 (0.18) | 0.34 | 0.80 (0.73, 0.89) | 0.88 (0.80, 0.96) | 0.95 (0.87, 1.04) |
| Mean diameter for LDL particles (nm) | 1466 | 24 (0.12) | 0.21 | 0.99 (0.90, 1.10) | 0.82 (0.74, 0.90) | 0.97 (0.89, 1.06) |
| Mean diameter for VLDL particles (nm) | 1466 | 37 (1.2) | 0.39 | 1.13 (1.03, 1.25) | 1.17 (1.07, 1.28) | 1.05 (0.97, 1.14) |
| Monounsaturated fatty acids 16:1, 18:1 (mmol/l) | 1462 | 2.8 (0.64) | 7.61 | 1.22 (1.11, 1.34) | 1.24 (1.13, 1.36) | 1.06 (0.97, 1.16) |
| Omega 3 fatty acids (mmol/l) | 1462 | 0.42 (0.11) | 6.91 | 1.11 (1.01, 1.22) | 1.23 (1.11, 1.35) | 0.99 (0.90, 1.09) |
| Omega 6 fatty acids (mmol/l) | 1462 | 2.7 (0.52) | 5.16 | 1.17 (1.06, 1.29) | 1.31 (1.18, 1.44) | 1.05 (0.96, 1.15) |
| Phenylalanine (mmol/l) | 1466 | 0.065 (0.01) | 6.77 | 1.06 (0.96, 1.17) | 0.97 (0.88, 1.06) | 1.08 (0.99, 1.18) |
| Phosphatidylcholine and other cholines (mmol/l) | 1463 | 1.5 (0.22) | 6.44 | 1.08 (0.98, 1.19) | 1.16 (1.05, 1.27) | 1.01 (0.93, 1.10) |
| Phospholipids in chylomicrons and extremely large VLDL (mmol/l) | 1466 | 0.0033 (0.0031) | 13.4 | 1.17 (1.07, 1.29) | 1.19 (1.09, 1.31) | 1.05 (0.97, 1.15) |
| Phospholipids in IDL (mmol/l) | 1466 | 0.21 (0.046) | 5.20 | 1.15 (1.04, 1.26) | 1.07 (0.98, 1.17) | 0.99 (0.90, 1.08) |
| Phospholipids in large HDL (mmol/l) | 1466 | 0.34 (0.091) | 3.56 | 0.80 (0.72, 0.88) | 0.90 (0.82, 0.99) | 0.98 (0.90, 1.06) |
| Phospholipids in large LDL (mmol/l) | 1466 | 0.24 (0.046) | 5.02 | 1.18 (1.07, 1.30) | 1.12 (1.02, 1.24) | 1.00 (0.92, 1.10) |
| Phospholipids in large VLDL (mmol/l) | 1466 | 0.048 (0.033) | 9.84 | 1.22 (1.11, 1.34) | 1.21 (1.10, 1.32) | 1.08 (0.99, 1.17) |
| Phospholipids in medium HDL (mmol/l) | 1466 | 0.35 (0.052) | 2.76 | 0.88 (0.80, 0.96) | 1.01 (0.93, 1.11) | 1.01 (0.93, 1.10) |
| Phospholipids in medium LDL (mmol/l) | 1466 | 0.15 (0.03) | 4.58 | 1.24 (1.12, 1.36) | 1.20 (1.09, 1.32) | 1.03 (0.95, 1.12) |
| Phospholipids in medium VLDL (mmol/l) | 1466 | 0.11 (0.048) | 7.13 | 1.27 (1.15, 1.39) | 1.22 (1.12, 1.34) | 1.08 (0.99, 1.18) |
| Phospholipids in small HDL (mmol/l) | 1466 | 0.56 (0.063) | 1.94 | 0.96 (0.88, 1.06) | 1.09 (1.00, 1.19) | 1.04 (0.96, 1.13) |
| Phospholipids in small LDL (mmol/l) | 1466 | 0.11 (0.021) | 7.38 | 1.21 (1.09, 1.33) | 1.22 (1.11, 1.34) | 1.02 (0.94, 1.11) |
| Phospholipids in small VLDL (mmol/l) | 1466 | 0.12 (0.031) | 4.36 | 1.28 (1.17, 1.41) | 1.19 (1.08, 1.30) | 1.06 (0.98, 1.16) |
| Phospholipids in very large HDL (mmol/l) | 1466 | 0.22 (0.073) | 4.49 | 0.84 (0.76, 0.93) | 0.91 (0.83, 0.99) | 0.97 (0.89, 1.05) |
| Phospholipids in very large VLDL (mmol/l) | 1466 | 0.011 (0.01) | 12.89 | 1.19 (1.09, 1.31) | 1.20 (1.09, 1.31) | 1.06 (0.97, 1.15) |
| Phospholipids in very small VLDL (mmol/l) | 1466 | 0.11 (0.026) | 4.70 | 1.19 (1.08, 1.31) | 1.10 (1.00, 1.20) | 1.02 (0.93, 1.11) |
| Phospholipids to total lipids ratio in chylomicrons and extremely large VLDL (%) | 1386 | 11 (2.1) | 5.12 | 1.00 (0.91, 1.10) | 1.13 (1.03, 1.24) | 1.01 (0.93, 1.10) |
| Phospholipids to total lipids ratio in IDL (%) | 1464 | 28 (0.92) | 1.04 | 0.82 (0.75, 0.91) | 0.85 (0.78, 0.94) | 0.95 (0.88, 1.04) |
| Phospholipids to total lipids ratio in large HDL (%) | 1465 | 49 (2.4) | 0.83 | 1.17 (1.06, 1.29) | 1.17 (1.07, 1.28) | 1.06 (0.97, 1.16) |
| Phospholipids to total lipids ratio in large LDL (%) | 1463 | 27 (2) | 1.02 | 0.90 (0.82, 0.99) | 0.95 (0.87, 1.03) | 0.97 (0.89, 1.06) |
| Phospholipids to total lipids ratio in large VLDL (%) | 1438 | 17 (0.88) | 1.08 | 1.23 (1.11, 1.35) | 1.20 (1.10, 1.31) | 1.06 (0.97, 1.15) |
| Phospholipids to total lipids ratio in medium HDL (%) | 1465 | 46 (1.4) | 0.72 | 1.23 (1.11, 1.36) | 0.90 (0.82, 1.00) | 1.05 (0.96, 1.14) |
| Phospholipids to total lipids ratio in medium LDL (%) | 1450 | 30 (3.8) | 1.23 | 1.01 (0.92, 1.11) | 1.00 (0.92, 1.09) | 1.00 (0.91, 1.09) |
| Phospholipids to total lipids ratio in medium VLDL (%) | 1465 | 20 (0.35) | 0.63 | 0.99 (0.90, 1.09) | 0.92 (0.85, 1.01) | 0.97 (0.89, 1.06) |
| Phospholipids to total lipids ratio in small HDL (%) | 1464 | 57 (3.5) | 1.17 | 0.93 (0.85, 1.03) | 1.00 (0.91, 1.10) | 0.99 (0.91, 1.09) |
| Phospholipids to total lipids ratio in small LDL (%) | 1449 | 34 (4.2) | 1.20 | 0.99 (0.90, 1.08) | 0.98 (0.89, 1.07) | 0.99 (0.91, 1.08) |
| Phospholipids to total lipids ratio in small VLDL (%) | 1464 | 24 (0.97) | 1.38 | 0.79 (0.72, 0.88) | 0.82 (0.75, 0.90) | 0.92 (0.85, 1.00) |
| Phospholipids to total lipids ratio in very large HDL (%) | 1465 | 47 (4.8) | 3.07 | 0.84 (0.76, 0.92) | 0.85 (0.77, 0.93) | 0.99 (0.90, 1.08) |
| Phospholipids to total lipids ratio in very large VLDL (%) | 1359 | 14 (2.7) | 4.25 | 1.14 (1.03, 1.26) | 1.18 (1.08, 1.30) | 0.97 (0.90, 1.06) |
| Phospholipids to total lipids ratio in very small VLDL (%) | 1465 | 27 (2.6) | 2.01 | 0.98 (0.89, 1.08) | 1.01 (0.92, 1.10) | 1.00 (0.92, 1.08) |
| Polyunsaturated fatty acids (mmol/l) | 1462 | 3.1 (0.6) | 5.22 | 1.17 (1.06, 1.29) | 1.31 (1.18, 1.44) | 1.04 (0.95, 1.14) |
| Ratio of 18:2 linoleic acid to total fatty acids (%) | 1462 | 24 (3.1) | 2.74 | 0.97 (0.88, 1.07) | 1.10 (1.00, 1.22) | 1.00 (0.92, 1.09) |
| Ratio of 22:6 docosahexaenoic acid to total fatty acids (%) | 1462 | 1.6 (0.23) | 5.48 | 0.84 (0.76, 0.92) | 0.89 (0.81, 0.98) | 0.86 (0.78, 0.94) |
| Ratio of apolipoprotein B to apolipoprotein A-I | 1465 | 0.51 (0.093) | 3.33 | 1.36 (1.24, 1.50) | 1.21 (1.10, 1.33) | 1.05 (0.96, 1.15) |
| Ratio of monounsaturated fatty acids to total fatty acids (%) | 1462 | 29 (1.9) | 2.68 | 1.18 (1.07, 1.30) | 1.06 (0.96, 1.16) | 1.05 (0.97, 1.15) |
| Ratio of omega 3 fatty acids to total fatty acids (%) | 1462 | 4.4 (0.64) | 2.95 | 0.91 (0.83, 1.01) | 1.02 (0.92, 1.12) | 0.89 (0.82, 0.98) |
| Ratio of omega 6 fatty acids to total fatty acids (%) | 1462 | 28 (2.4) | 1.89 | 0.92 (0.84, 1.02) | 1.05 (0.96, 1.15) | 1.01 (0.92, 1.10) |
| Ratio of polyunsaturated fatty acids to total fatty acids (%) | 1462 | 33 (2.5) | 1.65 | 0.90 (0.82, 0.99) | 1.05 (0.96, 1.15) | 0.98 (0.90, 1.07) |
| Ratio of saturated fatty acids to total fatty acids (%) | 1462 | 38 (1.5) | 2.23 | 0.94 (0.85, 1.04) | 0.84 (0.77, 0.93) | 0.96 (0.88, 1.05) |
| Ratio of triglycerides to phosphoglycerides | 1462 | 0.7 (0.23) | 6.96 | 1.22 (1.11, 1.34) | 1.20 (1.10, 1.31) | 1.06 (0.97, 1.16) |
| Remnant cholesterol non HDL non LDL cholesterol (mmol/l) | 1466 | 1.1 (0.26) | 6.61 | 1.27 (1.15, 1.39) | 1.20 (1.09, 1.32) | 1.04 (0.95, 1.13) |
| Saturated fatty acids (mmol/l) | 1462 | 3.6 (0.66) | 7.08 | 1.19 (1.09, 1.31) | 1.24 (1.13, 1.36) | 1.04 (0.95, 1.14) |
| Serum total cholesterol (mmol/l) | 1466 | 3.5 (0.59) | 4.29 | 1.12 (1.02, 1.24) | 1.14 (1.04, 1.25) | 1.01 (0.92, 1.10) |
| Serum total triglycerides (mmol/l) | 1466 | 1.2 (0.44) | 5.55 | 1.26 (1.15, 1.39) | 1.21 (1.10, 1.32) | 1.08 (0.99, 1.18) |
| Sphingomyelins (mmol/l) | 1463 | 0.3 (0.056) | 7.29 | 1.05 (0.96, 1.16) | 1.12 (1.03, 1.24) | 0.95 (0.87, 1.04) |
| Total cholesterol in chylomicrons and extremely large VLDL (mmol/l) | 1466 | 0.0046 (0.0039) | 12.87 | 1.21 (1.10, 1.33) | 1.18 (1.08, 1.29) | 1.05 (0.97, 1.15) |
| Total cholesterol in HDL (mmol/l) | 1466 | 1.3 (0.2) | 2.32 | 0.80 (0.73, 0.88) | 0.95 (0.87, 1.05) | 0.97 (0.89, 1.05) |
| Total cholesterol in HDL2 (mmol/l) | 1466 | 0.84 (0.19) | 3.38 | 0.79 (0.72, 0.87) | 0.95 (0.86, 1.04) | 0.97 (0.89, 1.05) |
| Total cholesterol in HDL3 (mmol/l) | 1466 | 0.48 (0.019) | 0.92 | 0.96 (0.87, 1.06) | 1.03 (0.94, 1.12) | 1.00 (0.92, 1.09) |
| Total cholesterol in IDL (mmol/l) | 1466 | 0.47 (0.12) | 7.02 | 1.15 (1.04, 1.26) | 1.09 (0.99, 1.20) | 0.99 (0.91, 1.08) |
| Total cholesterol in large HDL (mmol/l) | 1466 | 0.33 (0.11) | 4.10 | 0.79 (0.72, 0.87) | 0.88 (0.80, 0.97) | 0.97 (0.89, 1.05) |
| Total cholesterol in large LDL (mmol/l) | 1466 | 0.57 (0.16) | 6.51 | 1.16 (1.06, 1.28) | 1.11 (1.01, 1.22) | 1.00 (0.92, 1.10) |
| Total cholesterol in large VLDL (mmol/l) | 1466 | 0.06 (0.041) | 10.12 | 1.23 (1.12, 1.35) | 1.19 (1.09, 1.31) | 1.07 (0.98, 1.16) |
| Total cholesterol in LDL (mmol/l) | 1466 | 1.1 (0.33) | 6.14 | 1.16 (1.05, 1.27) | 1.12 (1.02, 1.23) | 1.00 (0.92, 1.10) |
| Total cholesterol in medium HDL (mmol/l) | 1466 | 0.37 (0.064) | 3.83 | 0.81 (0.74, 0.89) | 1.01 (0.93, 1.11) | 0.98 (0.91, 1.07) |
| Total cholesterol in medium LDL (mmol/l) | 1466 | 0.32 (0.11) | 6.14 | 1.16 (1.05, 1.27) | 1.13 (1.03, 1.23) | 1.01 (0.92, 1.11) |
| Total cholesterol in medium VLDL (mmol/l) | 1466 | 0.14 (0.062) | 8.48 | 1.27 (1.16, 1.40) | 1.21 (1.10, 1.32) | 1.07 (0.98, 1.17) |
| Total cholesterol in small HDL (mmol/l) | 1466 | 0.38 (0.053) | 4.27 | 0.99 (0.89, 1.09) | 1.06 (0.97, 1.16) | 1.02 (0.93, 1.12) |
| Total cholesterol in small LDL (mmol/l) | 1466 | 0.2 (0.066) | 5.40 | 1.14 (1.04, 1.25) | 1.12 (1.02, 1.23) | 1.00 (0.92, 1.10) |
| Total cholesterol in small VLDL (mmol/l) | 1466 | 0.18 (0.049) | 6.99 | 1.27 (1.16, 1.40) | 1.17 (1.06, 1.28) | 1.05 (0.96, 1.15) |
| Total cholesterol in very large HDL (mmol/l) | 1466 | 0.22 (0.058) | 6.47 | 0.90 (0.81, 0.99) | 0.99 (0.90, 1.08) | 0.96 (0.89, 1.05) |
| Total cholesterol in very large VLDL (mmol/l) | 1466 | 0.014 (0.012) | 12.15 | 1.20 (1.10, 1.32) | 1.18 (1.08, 1.29) | 1.05 (0.97, 1.15) |
| Total cholesterol in very small VLDL (mmol/l) | 1466 | 0.19 (0.042) | 6.48 | 1.17 (1.06, 1.28) | 1.06 (0.96, 1.16) | 0.99 (0.91, 1.09) |
| Total cholesterol in VLDL (mmol/l) | 1466 | 0.58 (0.18) | 6.71 | 1.28 (1.17, 1.41) | 1.21 (1.10, 1.33) | 1.06 (0.97, 1.16) |
| Total cholesterol to total lipids ratio in chylomicrons and extremely large VLDL (%) | 1386 | 16 (4.2) | 26.14 | 1.17 (1.05, 1.29) | 1.00 (0.89, 1.11) | 1.03 (0.95, 1.13) |
| Total cholesterol to total lipids ratio in IDL (%) | 1464 | 61 (2.6) | 1.44 | 0.93 (0.85, 1.02) | 1.06 (0.97, 1.16) | 0.95 (0.88, 1.04) |
| Total cholesterol to total lipids ratio in large HDL (%) | 1465 | 48 (2.3) | 1.13 | 0.77 (0.70, 0.84) | 0.85 (0.78, 0.94) | 0.91 (0.84, 1.00) |
| Total cholesterol to total lipids ratio in large LDL (%) | 1463 | 64 (3.7) | 0.99 | 1.00 (0.91, 1.10) | 1.06 (0.97, 1.17) | 0.99 (0.91, 1.08) |
| Total cholesterol to total lipids ratio in large VLDL (%) | 1438 | 21 (2.5) | 3.38 | 1.13 (1.02, 1.25) | 1.04 (0.95, 1.14) | 0.98 (0.90, 1.06) |
| Total cholesterol to total lipids ratio in medium HDL (%) | 1465 | 49 (2.4) | 0.86 | 0.76 (0.69, 0.83) | 0.99 (0.91, 1.09) | 0.94 (0.87, 1.02) |
| Total cholesterol to total lipids ratio in medium LDL (%) | 1450 | 63 (5.3) | 0.98 | 0.95 (0.87, 1.05) | 1.01 (0.93, 1.11) | 0.98 (0.90, 1.07) |
| Total cholesterol to total lipids ratio in medium VLDL (%) | 1465 | 24 (2.5) | 3.76 | 1.08 (0.98, 1.20) | 0.99 (0.90, 1.08) | 1.00 (0.92, 1.09) |
| Total cholesterol to total lipids ratio in small HDL (%) | 1464 | 39 (4.1) | 1.82 | 0.99 (0.90, 1.09) | 0.98 (0.89, 1.07) | 0.99 (0.91, 1.08) |
| Total cholesterol to total lipids ratio in small LDL (%) | 1449 | 59 (5.9) | 1.02 | 0.96 (0.87, 1.05) | 0.99 (0.91, 1.09) | 0.98 (0.90, 1.08) |
| Total cholesterol to total lipids ratio in small VLDL (%) | 1464 | 34 (4.2) | 3.49 | 0.93 (0.84, 1.03) | 0.93 (0.85, 1.02) | 0.96 (0.88, 1.05) |
| Total cholesterol to total lipids ratio in very large HDL (%) | 1465 | 49 (4) | 2.69 | 1.14 (1.03, 1.25) | 1.18 (1.08, 1.30) | 0.99 (0.90, 1.08) |
| Total cholesterol to total lipids ratio in very large VLDL (%) | 1359 | 20 (2.7) | 4.99 | 1.13 (1.02, 1.26) | 0.98 (0.89, 1.08) | 0.99 (0.91, 1.08) |
| Total cholesterol to total lipids ratio in very small VLDL (%) | 1465 | 49 (3.1) | 2.81 | 0.83 (0.75, 0.91) | 0.87 (0.80, 0.96) | 0.90 (0.82, 0.98) |
| Total cholines (mmol/l) | 1463 | 1.9 (0.25) | 5.84 | 1.08 (0.99, 1.19) | 1.17 (1.07, 1.29) | 1.02 (0.94, 1.11) |
| Total fatty acids (mmol/l) | 1462 | 9.6 (1.8) | 6.06 | 1.20 (1.09, 1.32) | 1.27 (1.16, 1.40) | 1.05 (0.96, 1.15) |
| Total lipids in chylomicrons and extremely large VLDL (mmol/l) | 1466 | 0.028 (0.024) | 13.08 | 1.18 (1.07, 1.29) | 1.20 (1.09, 1.31) | 1.05 (0.97, 1.15) |
| Total lipids in IDL (mmol/l) | 1466 | 0.77 (0.17) | 5.96 | 1.18 (1.07, 1.29) | 1.09 (1.00, 1.20) | 1.00 (0.92, 1.09) |
| Total lipids in large HDL (mmol/l) | 1466 | 0.69 (0.21) | 3.68 | 0.80 (0.72, 0.88) | 0.89 (0.81, 0.98) | 0.97 (0.89, 1.06) |
| Total lipids in large LDL (mmol/l) | 1466 | 0.88 (0.22) | 5.81 | 1.18 (1.07, 1.30) | 1.11 (1.01, 1.22) | 1.01 (0.92, 1.10) |
| Total lipids in large VLDL (mmol/l) | 1466 | 0.28 (0.18) | 9.46 | 1.22 (1.11, 1.34) | 1.21 (1.10, 1.32) | 1.07 (0.99, 1.17) |
| Total lipids in medium HDL (mmol/l) | 1466 | 0.76 (0.12) | 3.29 | 0.85 (0.78, 0.94) | 1.03 (0.94, 1.12) | 1.00 (0.92, 1.09) |
| Total lipids in medium LDL (mmol/l) | 1466 | 0.5 (0.14) | 5.34 | 1.19 (1.08, 1.31) | 1.14 (1.04, 1.26) | 1.02 (0.93, 1.12) |
| Total lipids in medium VLDL (mmol/l) | 1466 | 0.56 (0.25) | 7.27 | 1.26 (1.15, 1.39) | 1.22 (1.12, 1.34) | 1.08 (0.99, 1.18) |
| Total lipids in small HDL (mmol/l) | 1466 | 0.99 (0.089) | 2.69 | 1.00 (0.90, 1.10) | 1.12 (1.02, 1.22) | 1.05 (0.97, 1.15) |
| Total lipids in small LDL (mmol/l) | 1466 | 0.33 (0.088) | 5.64 | 1.18 (1.07, 1.30) | 1.16 (1.06, 1.27) | 1.02 (0.93, 1.11) |
| Total lipids in small VLDL (mmol/l) | 1466 | 0.52 (0.14) | 5.23 | 1.30 (1.19, 1.44) | 1.21 (1.10, 1.33) | 1.07 (0.98, 1.17) |
| Total lipids in very large HDL (mmol/l) | 1466 | 0.46 (0.13) | 4.87 | 0.87 (0.79, 0.96) | 0.95 (0.86, 1.04) | 0.97 (0.89, 1.05) |
| Total lipids in very large VLDL (mmol/l) | 1466 | 0.071 (0.059) | 11.56 | 1.19 (1.08, 1.30) | 1.19 (1.09, 1.30) | 1.06 (0.97, 1.16) |
| Total lipids in very small VLDL (mmol/l) | 1466 | 0.39 (0.08) | 4.72 | 1.24 (1.13, 1.37) | 1.11 (1.01, 1.22) | 1.02 (0.94, 1.12) |
| Total phosphoglycerides (mmol/l) | 1463 | 1.7 (0.23) | 5.15 | 1.09 (0.99, 1.19) | 1.17 (1.07, 1.29) | 1.02 (0.94, 1.12) |
| Triglycerides in chylomicrons and extremely large VLDL (mmol/l) | 1466 | 0.02 (0.017) | 13.44 | 1.17 (1.07, 1.29) | 1.20 (1.10, 1.31) | 1.05 (0.97, 1.15) |
| Triglycerides in HDL (mmol/l) | 1466 | 0.12 (0.031) | 4.25 | 1.20 (1.09, 1.32) | 1.08 (0.99, 1.19) | 1.07 (0.99, 1.17) |
| Triglycerides in IDL (mmol/l) | 1466 | 0.083 (0.022) | 3.66 | 1.29 (1.17, 1.41) | 1.06 (0.97, 1.16) | 1.06 (0.97, 1.15) |
| Triglycerides in large HDL (mmol/l) | 1466 | 0.022 (0.011) | 6.02 | 1.03 (0.93, 1.14) | 0.92 (0.83, 1.02) | 1.03 (0.95, 1.13) |
| Triglycerides in large LDL (mmol/l) | 1466 | 0.069 (0.019) | 4.10 | 1.27 (1.15, 1.40) | 1.04 (0.95, 1.15) | 1.05 (0.97, 1.15) |
| Triglycerides in large VLDL (mmol/l) | 1466 | 0.17 (0.11) | 9.25 | 1.21 (1.10, 1.33) | 1.21 (1.10, 1.32) | 1.08 (0.99, 1.17) |
| Triglycerides in LDL (mmol/l) | 1466 | 0.13 (0.034) | 3.80 | 1.27 (1.15, 1.39) | 1.07 (0.98, 1.18) | 1.06 (0.98, 1.16) |
| Triglycerides in medium HDL (mmol/l) | 1466 | 0.038 (0.0098) | 6.07 | 1.20 (1.09, 1.32) | 1.13 (1.04, 1.24) | 1.08 (0.99, 1.17) |
| Triglycerides in medium LDL (mmol/l) | 1466 | 0.034 (0.0098) | 4.08 | 1.26 (1.15, 1.39) | 1.09 (0.99, 1.19) | 1.07 (0.98, 1.16) |
| Triglycerides in medium VLDL (mmol/l) | 1466 | 0.31 (0.14) | 7.15 | 1.25 (1.14, 1.37) | 1.23 (1.12, 1.34) | 1.08 (0.99, 1.18) |
| Triglycerides in small HDL (mmol/l) | 1466 | 0.043 (0.011) | 3.87 | 1.28 (1.16, 1.40) | 1.14 (1.05, 1.25) | 1.09 (1.00, 1.19) |
| Triglycerides in small LDL (mmol/l) | 1466 | 0.024 (0.0068) | 10.58 | 1.30 (1.18, 1.43) | 1.21 (1.10, 1.32) | 1.08 (1.00, 1.18) |
| Triglycerides in small VLDL (mmol/l) | 1466 | 0.22 (0.074) | 5.54 | 1.30 (1.18, 1.43) | 1.22 (1.11, 1.33) | 1.09 (1.00, 1.19) |
| Triglycerides in very large HDL (mmol/l) | 1466 | 0.015 (0.0064) | 7.39 | 1.15 (1.04, 1.26) | 1.07 (0.98, 1.18) | 1.05 (0.96, 1.15) |
| Triglycerides in very large VLDL (mmol/l) | 1466 | 0.046 (0.037) | 11.26 | 1.18 (1.08, 1.30) | 1.19 (1.09, 1.31) | 1.06 (0.98, 1.16) |
| Triglycerides in very small VLDL (mmol/l) | 1466 | 0.09 (0.023) | 4.42 | 1.31 (1.19, 1.44) | 1.16 (1.06, 1.27) | 1.08 (0.99, 1.18) |
| Triglycerides in VLDL (mmol/l) | 1466 | 0.87 (0.39) | 6.75 | 1.25 (1.13, 1.37) | 1.22 (1.11, 1.34) | 1.08 (0.99, 1.18) |
| Triglycerides to total lipids ratio in chylomicrons and extremely large VLDL (%) | 1386 | 73 (4.9) | 6.40 | 0.87 (0.79, 0.96) | 0.94 (0.86, 1.04) | 0.97 (0.89, 1.06) |
| Triglycerides to total lipids ratio in IDL (%) | 1464 | 11 (2.8) | 5.90 | 1.14 (1.03, 1.25) | 1.00 (0.91, 1.09) | 1.06 (0.98, 1.16) |
| Triglycerides to total lipids ratio in large HDL (%) | 1465 | 3.2 (1.2) | 6.44 | 1.28 (1.16, 1.41) | 1.01 (0.92, 1.11) | 1.07 (0.98, 1.16) |
| Triglycerides to total lipids ratio in large LDL (%) | 1463 | 8.2 (2.4) | 5.27 | 1.09 (0.99, 1.20) | 0.95 (0.86, 1.04) | 1.04 (0.96, 1.13) |
| Triglycerides to total lipids ratio in large VLDL (%) | 1438 | 62 (3.3) | 1.33 | 0.86 (0.78, 0.95) | 0.92 (0.84, 1.01) | 1.00 (0.92, 1.09) |
| Triglycerides to total lipids ratio in medium HDL (%) | 1465 | 5 (1.4) | 4.94 | 1.30 (1.19, 1.43) | 1.10 (1.01, 1.20) | 1.06 (0.97, 1.15) |
| Triglycerides to total lipids ratio in medium LDL (%) | 1450 | 7.1 (2) | 5.98 | 1.11 (1.01, 1.22) | 0.96 (0.88, 1.06) | 1.05 (0.96, 1.14) |
| Triglycerides to total lipids ratio in medium VLDL (%) | 1465 | 56 (2.7) | 1.91 | 0.93 (0.84, 1.03) | 1.02 (0.93, 1.12) | 1.00 (0.92, 1.09) |
| Triglycerides to total lipids ratio in small HDL (%) | 1464 | 4.4 (1.1) | 2.91 | 1.27 (1.16, 1.40) | 1.09 (1.00, 1.20) | 1.06 (0.98, 1.16) |
| Triglycerides to total lipids ratio in small LDL (%) | 1449 | 7.4 (2.1) | 4.82 | 1.16 (1.05, 1.27) | 1.07 (0.98, 1.17) | 1.06 (0.98, 1.16) |
| Triglycerides to total lipids ratio in small VLDL (%) | 1464 | 42 (4.4) | 2.51 | 1.13 (1.02, 1.24) | 1.11 (1.02, 1.22) | 1.06 (0.97, 1.15) |
| Triglycerides to total lipids ratio in very large HDL (%) | 1465 | 3.4 (1.4) | 9.72 | 1.26 (1.15, 1.39) | 1.12 (1.02, 1.22) | 1.07 (0.98, 1.17) |
| Triglycerides to total lipids ratio in very large VLDL (%) | 1359 | 66 (4.3) | 2.21 | 0.84 (0.76, 0.94) | 0.91 (0.83, 1.00) | 1.02 (0.94, 1.12) |
| Triglycerides to total lipids ratio in very small VLDL (%) | 1465 | 24 (4.8) | 4.16 | 1.14 (1.03, 1.25) | 1.09 (0.99, 1.19) | 1.07 (0.98, 1.16) |
| Tyrosine (mmol/l) | 1462 | 0.053 (0.011) | 8.61 | 1.02 (0.92, 1.12) | 1.07 (0.97, 1.17) | 1.06 (0.98, 1.15) |
| Valine (mmol/l) | 1464 | 0.17 (0.037) | 5.82 | 1.03 (0.94, 1.14) | 1.09 (1.00, 1.20) | 0.92 (0.85, 1.01) |
| 18:2 linoleic acid (mmol/l) | 1462 | 2.3 (0.51) | 5.34 | 1.17 (1.06, 1.29) | 1.31 (1.18, 1.45) | 1.04 (0.95, 1.14) |
| 22:6 docosahexaenoic acid (mmol/l) | 1462 | 0.15 (0.032) | 8.14 | 1.05 (0.95, 1.15) | 1.16 (1.06, 1.28) | 0.94 (0.86, 1.04) |
| β-hydroxybutyrate (mmol/l) | 1437 | 0.1 (0.033) | 4.98 | 1.35 (1.18, 1.54) | 1.21 (1.08, 1.36) | 1.22 (1.08, 1.36) |

**Legend** Odds ratios (OR) adjusted for age, sex, fasting time, region, smoking status, educational attainment and triglycerides. Values represent effect estimate per one standard deviation higher metabolic marker. Coefficients of variation (CV) were calculated in 137 duplicate measures; SD: standard deviation.

# Online Table 3. Adjusted ORs (95% CI) of myocardial infarction for traits measured by clinical chemistry and NMR spectroscopy

| **Trait** | **Number**  **(Cases/Controls)** | **Clinical chemistry**  **OR (95% CI)** | **NMR spectroscopy**  **OR (95% CI)** |
| --- | --- | --- | --- |
|  |  |  |  |
| Total cholesterol | 912/1466 | 1.23 (1.12, 1.36) | 1.12 (1.02, 1.24) |
| LDL-cholesterol | 912/1466 | 1.25 (1.13, 1.37) | 1.16 (1.04, 1.27) |
| HDL-cholesterol | 912/1466 | 0.84 (0.76, 0.93) | 0.80 (0.73, 0.88) |
| Triglycerides | 912/1466 | 1.15 (1.05, 1.26) | 1.26 (1.15, 1.38) |
| Apolipoprotein B | 912/1466 | 1.29 (1.17, 1.41) | 1.30 (1.18, 1.43) |
| Apolipoprotein A-1 | 912/1466 | 0.79 (0.72, 0.88) | 0.89 (0.81, 0.98) |
| Creatinine | 912/1466 | 1.45 (1.13, 1.86) | 1.38 (1.09, 1.74) |
| Albumin | 912/1466 | 0.86 (0.78, 0.95) | 0.88 (0.80, 0.98) |

**Legend** Odds ratios (OR) adjusted for age, sex, fasting time, region, smoking status and educational attainment. Values represent effect estimate per one standard deviation higher trait.

# Online Table 4. Adjusted ORs (95% CI) of myocardial infarction and ischemic stroke for fatty acids additionally adjusted for triglycerides

| Fatty acid adjusted for TG | Myocardial infarction  OR (95% CI) | P-value | Ischemic stroke  OR (95% CI) | P-value |
| --- | --- | --- | --- | --- |
| Total fatty acids | 0.98 (0.84, 1.15) | 0.88 | 1.28 (1.09, 1.50) | 0.02 |
| Omega 6 fatty acids | 1.04 (0.93, 1.17) | 0.55 | 1.25 (1.12, 1.40) | 2.82x10^-3^ |
| Linoleic acid | 1.05 (0.94, 1.18) | 0.43 | 1.25 (1.11, 1.40) | 2.82x10^-3^ |
| Omega 3 fatty acids | 0.91 (0.80, 1.03) | 0.20 | 1.13 (0.99, 1.29) | 0.16 |
| Docosahexaenoic acid | 0.90 (0.81, 1.01) | 0.14 | 1.08 (0.97, 1.20) | 0.34 |
| Polyunsaturated fatty acids | 1.02 (0.91, 1.15) | 0.76 | 1.25 (1.11, 1.41) | 2.95x10^-3^ |
| Monounsaturated fatty acids | 0.93 (0.75, 1.16) | 0.60 | 1.26 (1.01, 1.58) | 0.10 |
| Saturated fatty acids | 0.94 (0.80, 1.12) | 0.57 | 1.20 (1.01, 1.42) | 0.10 |

**Legend** Odds ratios (OR) adjusted for age, sex, fasting time, region, smoking status, educational attainment and triglycerides. Values represent effect estimate per one standard deviation higher fatty acid.

# Online Table 5. Discriminatory ability

|  | **Area under the Receiver Operating Characteristic curve** (95%CI) | | |
| --- | --- | --- | --- |
| Vascular endpoint | **Model 1** (age, sex, smoking, systolic blood pressure, body mass index, type 2 diabetes and total cholesterol/HDL-cholesterol) | **Model 2** (Model 1 + 18 principal components explaining 95% variance of 225 metabolic markers) | P-value for difference between Models 2 and 1 |
| **Myocardial infarction** | 0.800 (0.782, 0.819) | 0.813 (0.795, 0.832) | 7.30x10^-5^ |
| **Ischemic stroke** | 0.767 (0.748, 0.786) | 0.775 (0.757, 0.794) | 9.09x10^-3^ |
| **Intracerebral hemorrhage** | 0.797 (0.778, 0.815) | 0.809 (0.791, 0.827) | 2.98x10^-4^ |

#
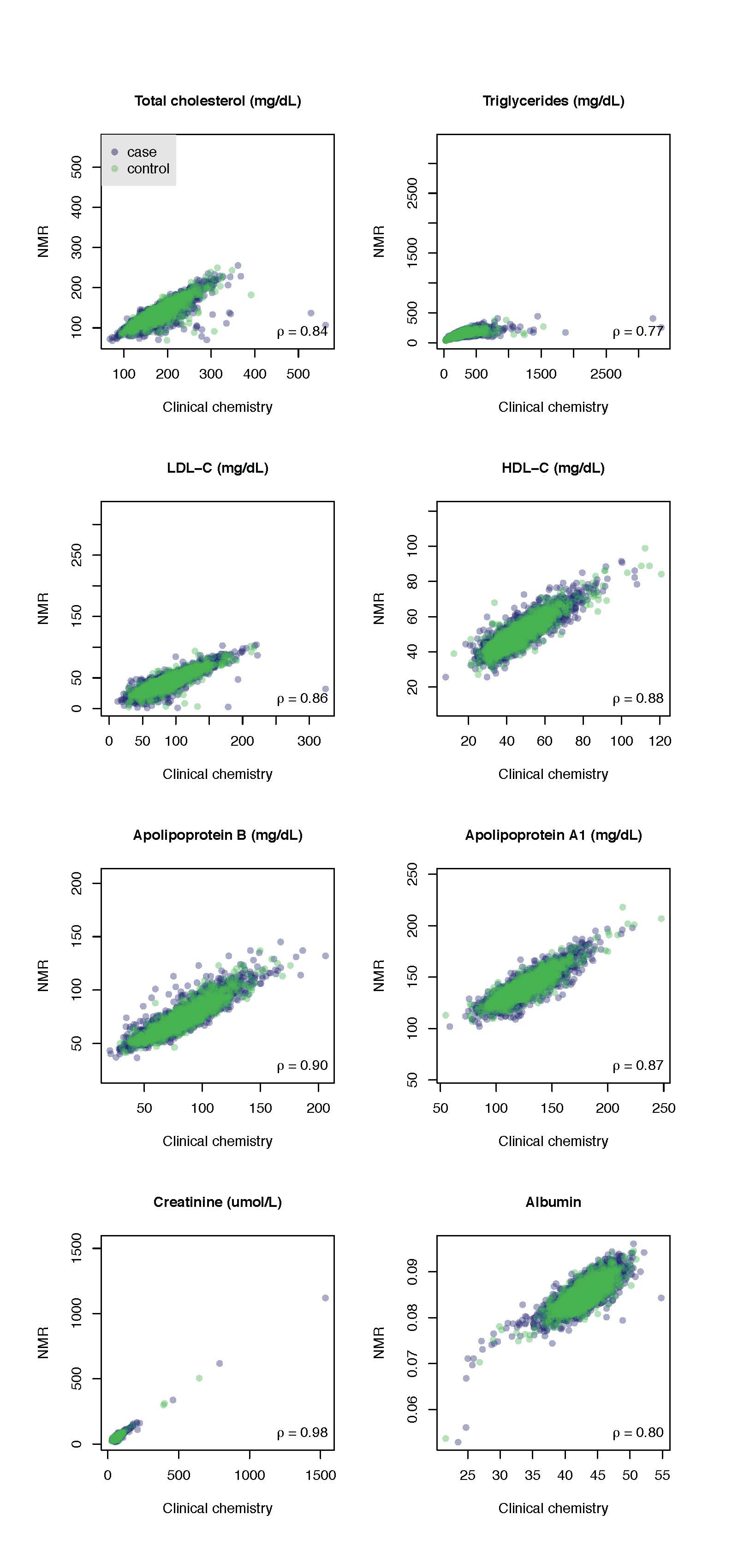
Online Figure 1. Scatterplots of 8 traits measured by both clinical chemistry and NMR spectroscopy

# Online Figure 2. Adjusted ORs (95% CI) of myocardial infarction for lipoprotein particle concentration, cholesterol and triglycerides, with additional adjustment for SBP and BMI


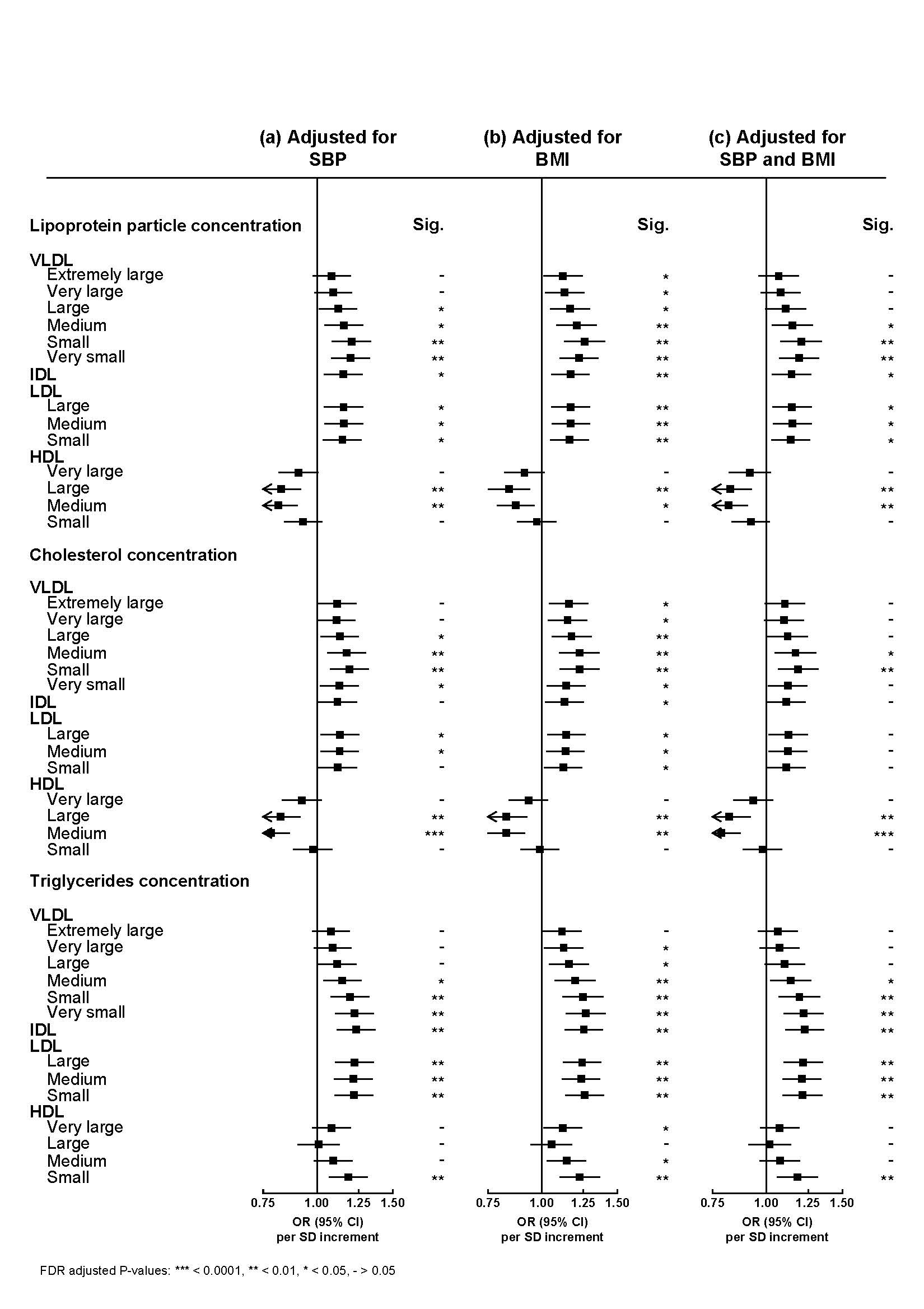


# Online Figure 3. Adjusted ORs (95% CI) of myocardial infarction for particle diameter, cholesterol and triglycerides, with additional adjustment for SBP and BMI


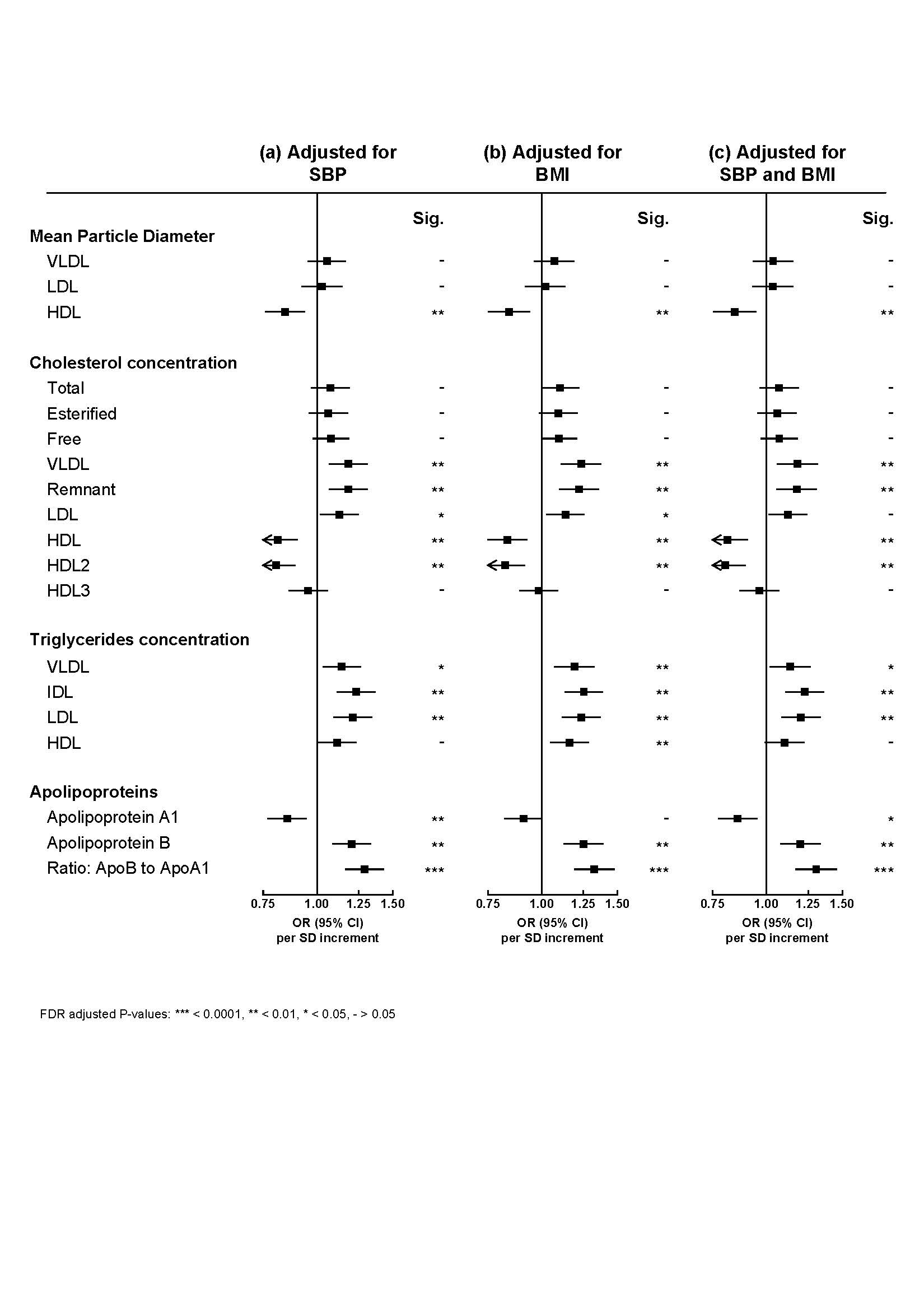


# Online Figure 4. Adjusted ORs (95% CI) of myocardial infarction for other traits, with additional adjustment for SBP and BMI


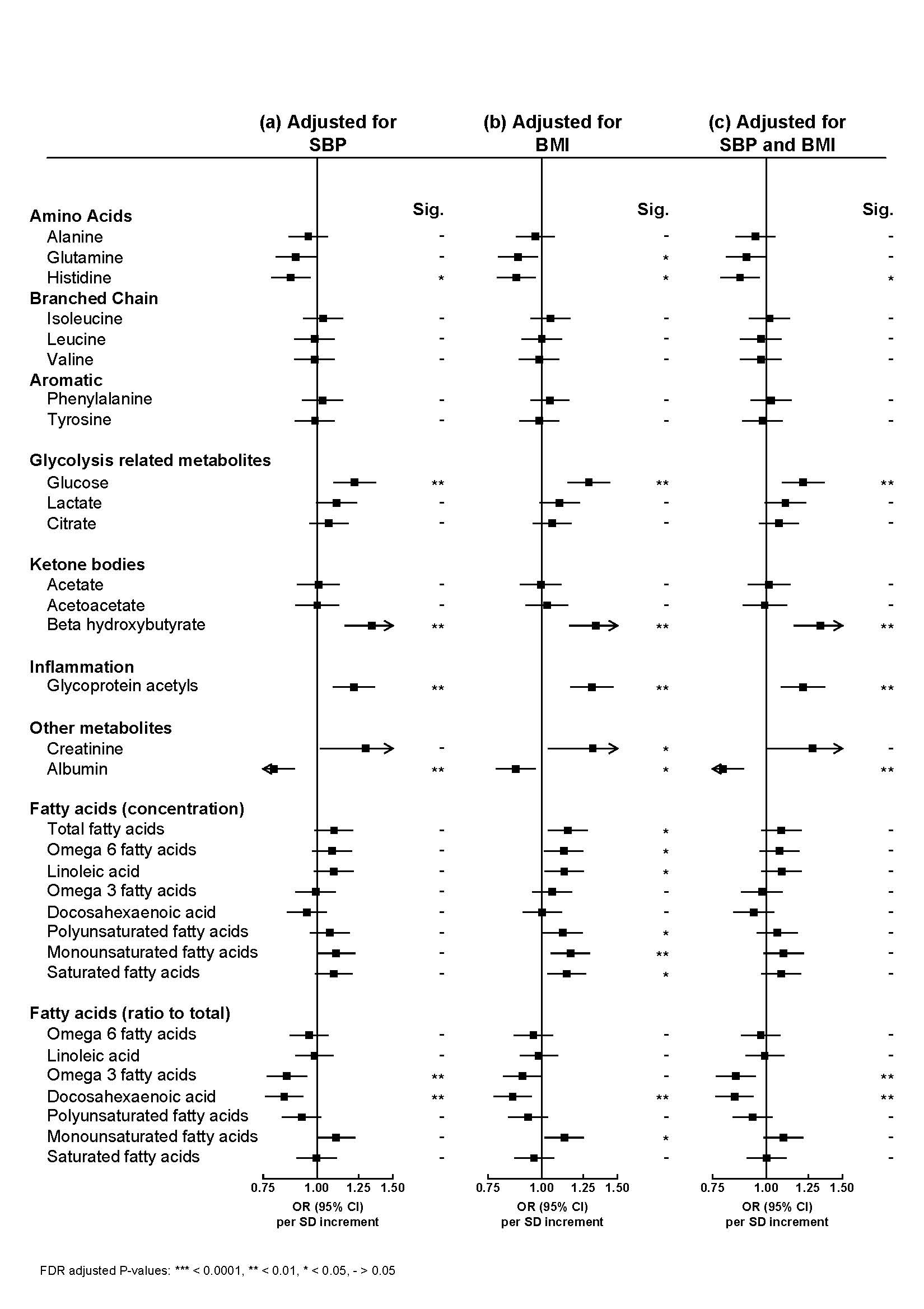


# Online Figure 5. Adjusted ORs (95% CI) of ischemic stroke for lipoprotein particle concentration, cholesterol and triglycerides, with additional adjustment for SBP and BMI


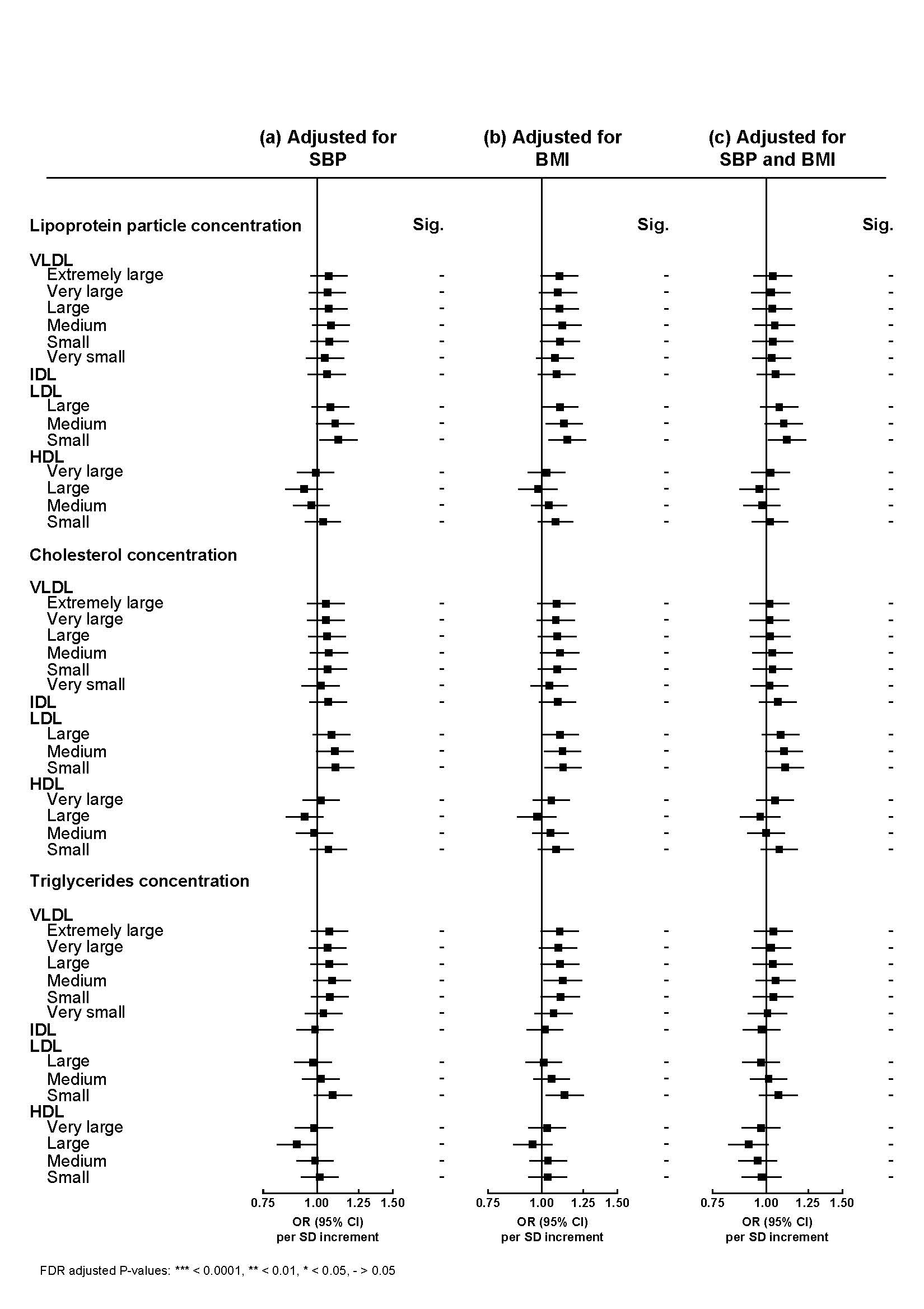


# Online Figure 6. Adjusted ORs (95% CI) of ischemic stroke for particle diameter, cholesterol and triglycerides, with additional adjustment for SBP and BMI


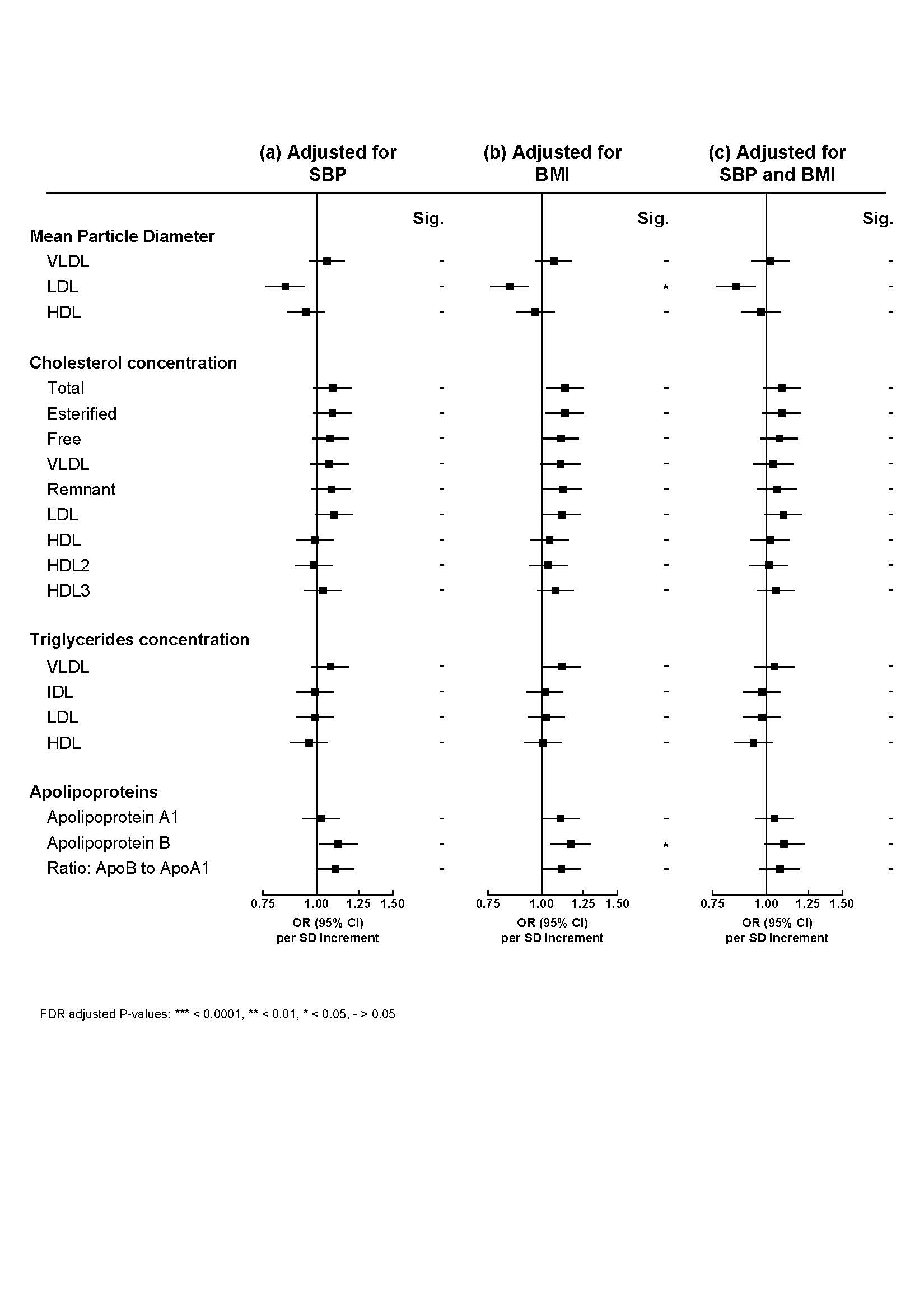


# Online Figure 7. Adjusted ORs (95% CI) of ischemic stroke for other traits, with additional adjustment for SBP and BMI


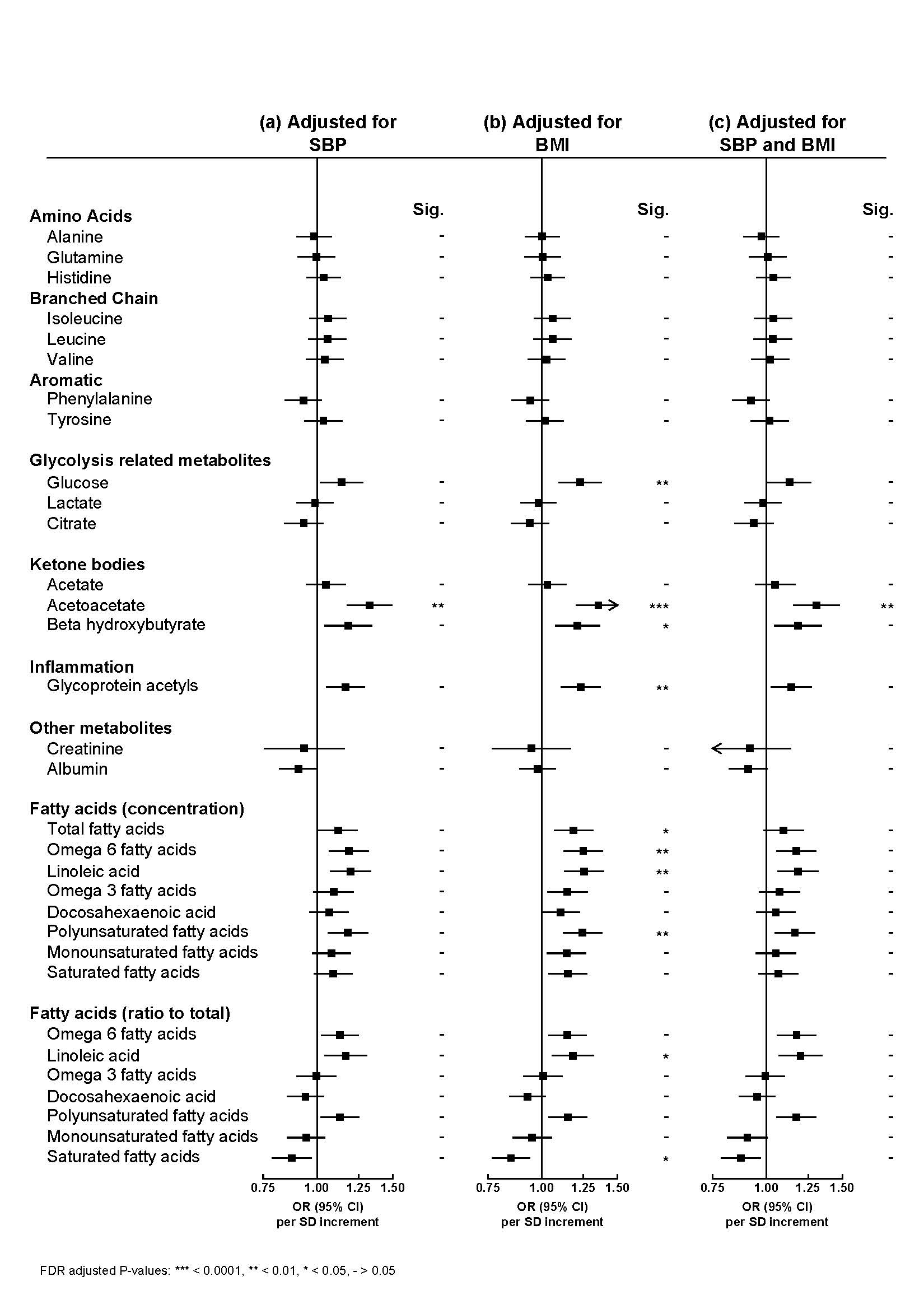


# Online Figure 8. Adjusted ORs (95% CI) of intracerebral hemorrhage for lipoprotein particle concentration, cholesterol and triglycerides, with additional adjustment for SBP and BMI


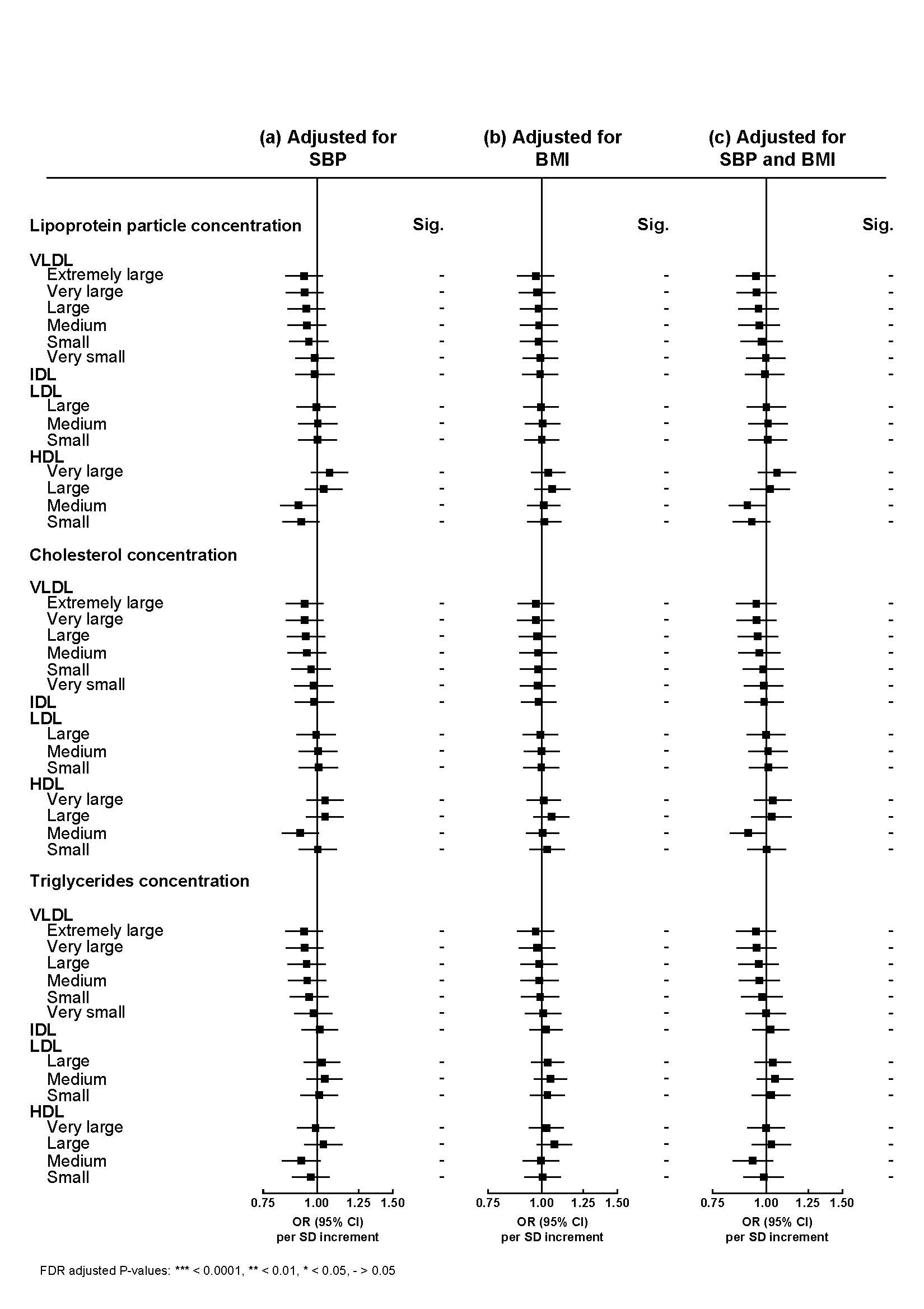


# Online Figure 9. Adjusted ORs (95% CI) of intracerebral hemorrhage for particle diameter, cholesterol and triglycerides, with additional adjustment for SBP and BMI


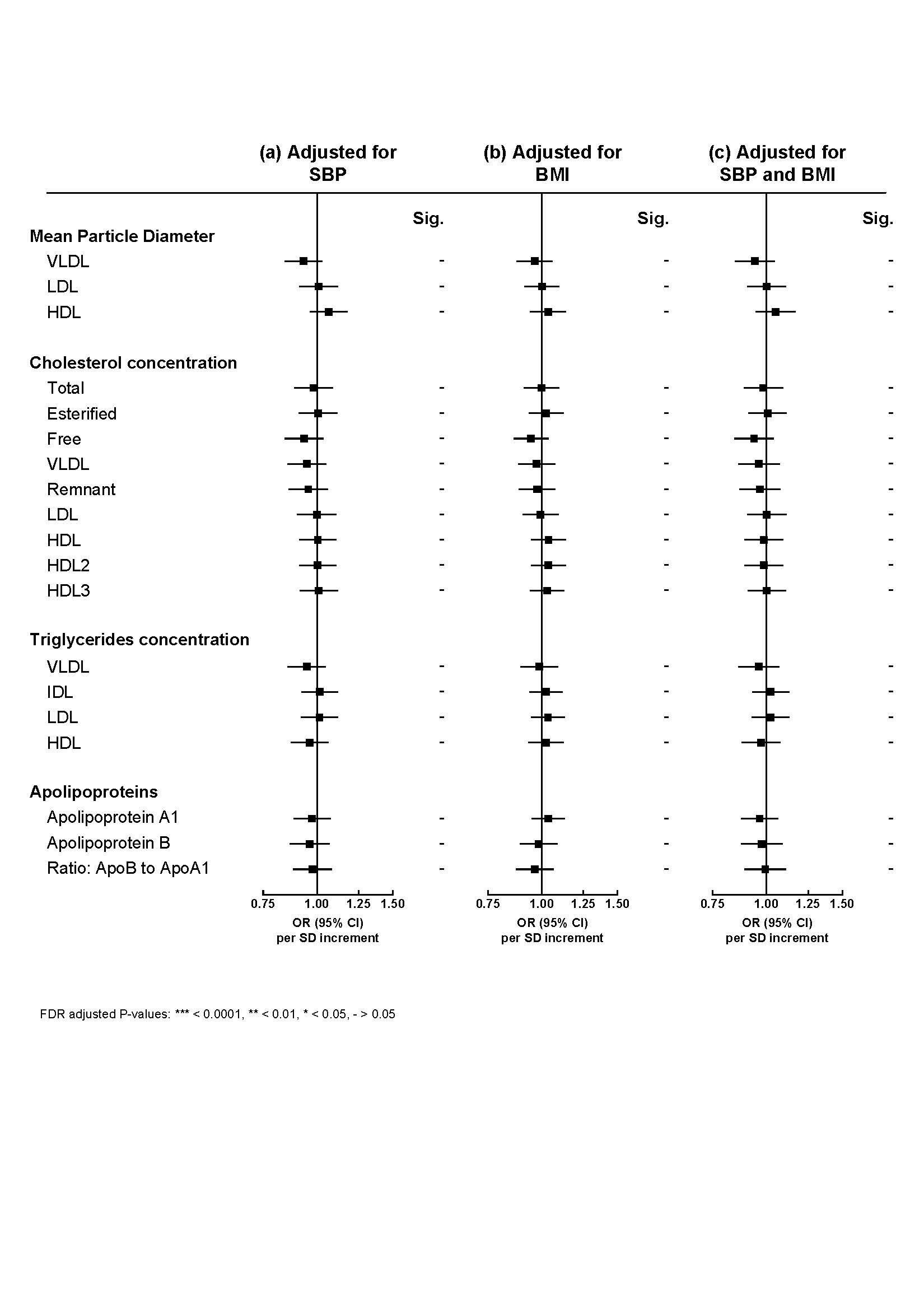


# Online Figure 10. Adjusted ORs (95% CI) of intracerebral hemorrhage for other traits, with additional adjustment for SBP and BMI


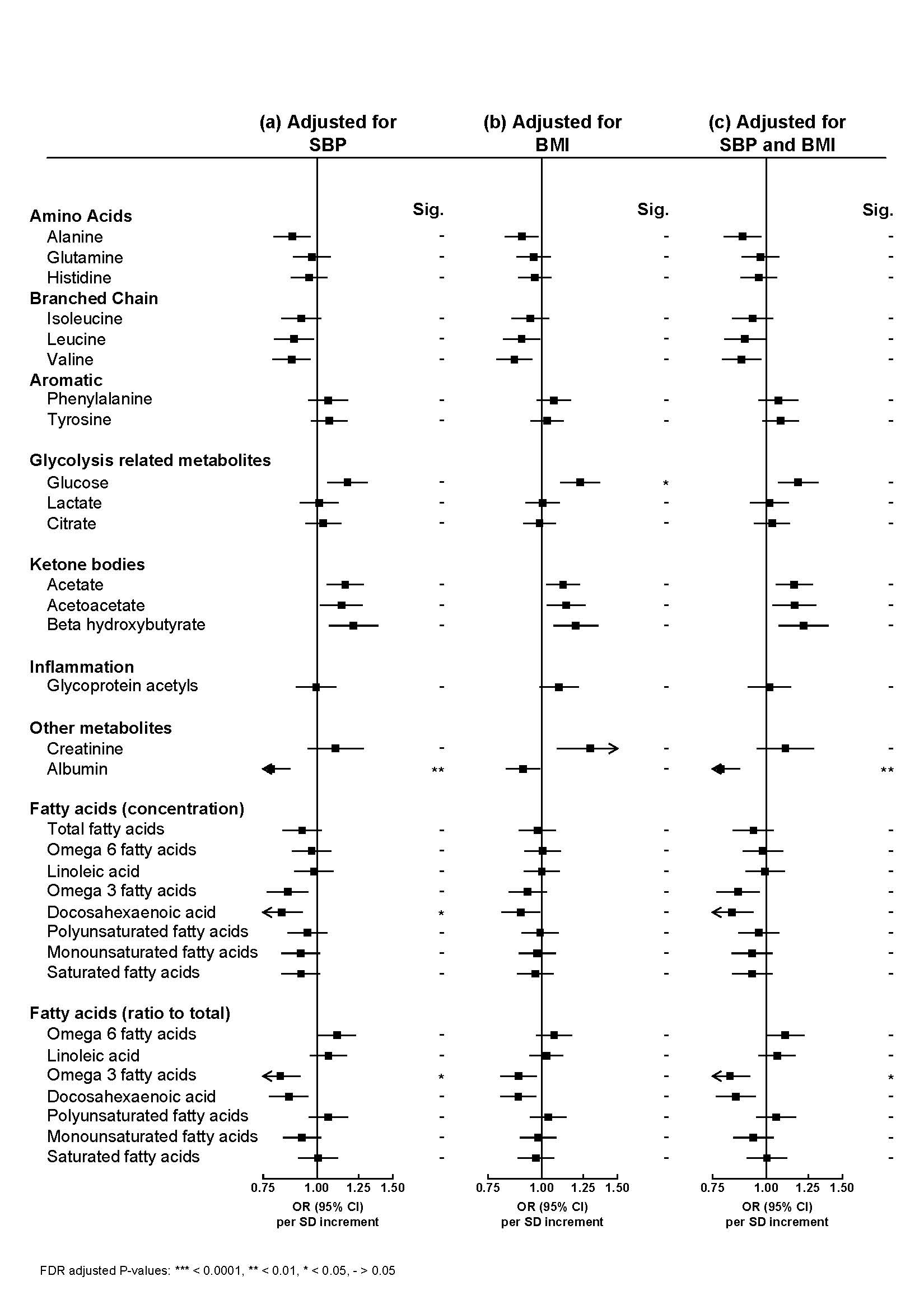

Supplement: Online Tables 1–5 and Online Figures 1–10 [file mmc1.docx]
